# Characterization With KRAS Mutant Is a Critical Determinant in Immunotherapy and Other Multiple Therapies for Non-Small Cell Lung Cancer

**DOI:** 10.3389/fonc.2021.780655

**Published:** 2022-01-05

**Authors:** Mo Shen, Rongbin Qi, Justin Ren, Dongqing Lv, Haihua Yang

**Affiliations:** ^1^ Key Laboratory of Radiation Oncology of Taizhou, Radiation Oncology Institute of Enze Medical Health Academy, Affiliated Taizhou Hospital of Wenzhou Medical University, Taizhou, China; ^2^ The First Clinical Medical College of Zhejiang Chinese Medical University, Hangzhou, China; ^3^ Department of Respiratory Medicine, Enze Hospital, Affiliated Taizhou Hospital of Wenzhou Medical University, Taizhou, China; ^4^ Biological Sciences, Northwestern University, Evanston, Evanston, IL, United States; ^5^ Department of Radiation Oncology, Enze Hospital, Affiliated Taizhou Hospital of Wenzhou Medical University, Taizhou, China

**Keywords:** KRAS, NSCLC, chemotherapy, immunotherapy, targeted therapy

## Abstract

Non-small cell lung cancer (NSCLC) is a frequent type of cancer, which is mainly characterized clinically by high aggressiveness and high mortality. KRAS oncoprotein is the most common molecular protein detected in NSCLC, accounting for 25% of all oncogenic mutations. Constitutive activation of the KRAS oncoprotein triggers an intracellular cascade in cancer cells, leading to uncontrolled cell proliferation of cancer cells and aberrant cell survival states. The results of multiple clinical trials have shown that different KRAS mutation subtypes exhibit different sensitivities to different chemotherapy regimens. Meanwhile, anti-angiogenic drugs have shown differential efficacy for different subtypes of KRAS mutated lung cancer. It was explored to find if the specificity of the KRAS mutation subtype would affect PD-L1 expression, so immunotherapy would be of potential clinical value for the treatment of some types of KRAS mutations. It was discovered that the specificity of the KRAS mutation affected PD-L1, which opened up immunotherapy as a potential clinical treatment option. After several breakthrough studies, the preliminary test data of many early clinical trials showed that it is possible to directly inhibit KRAS G12C mutation, which has been proved to be a targeted treatment that is suitable for about 10%–12% of patients with advanced NSCLC, having a significant impact on the prolongation of their survival and the improvement of their quality of life. This article reviews the latest progress of treatments for NSCLC with KRAS mutation, in order to gain insight into the biological diversity of lung cancer cells and their potential clinical implications, thereby enabling individualized treatment for patients with KRAS-mutant NSCLC.

## Background

Lung cancer is one of the leading causes of cancer death in the world with 1.8 million deaths every year. The 5-year survival rate for patients with lung cancer is approximately 20% (1). Non-small cell lung cancer (NSCLC) accounts for 80%–85% of the total number of lung cancer cases (2). Through recent research, there has been great advancement in the treatment of NSCLC patients with epidermal growth factor receptor (EGFR) mutation and anaplastic lymphoma receptor tyrosine kinase (ALK) rearrangement (3–6). However, effective treatments for kirsten rat sarcoma viral oncogene homolog (KRAS) mutations have not been developed. KRAS mutations are found in 25%–50% of Caucasian NSCLC patients and 5%–10% of Asian NSCLC patients (7–10). In patients with stage IV NSCLC, the results of platinum-based chemotherapy as the first form of treatment are very poor. It is obviously necessary to improve the treatment methods and provide individualized treatment for each patient (11). The NSCLC molecular spectrum is the key factor in treatment decision-making. There are many emerging carcinogenic targets and active targeted drugs. Somatic mutation of EGFR and rearrangement of ALK, proto-oncogene tyrosine protein kinase (ROS1), and proto-oncogene (RET) are supposed to be dependable biomarkers and effective drug targets for NSCLC (12, 13). However, the rat sarcoma viral oncogene homolog (RAS) family is the most common mutated oncogene, yet this oncogene has been defined as untreatable. Despite more than 40 years of basic and clinical research, there is still no effective anti-RAS therapy in the actual clinical diagnosis and treatment process. In recent years, targeted therapy and immunotherapy have been booming. At the same time, direct KRAS targeting and KRAS-related immunotherapy have also made great progress (14, 15). This paper will look to review the biological basis of KRAS mutations in NSCLC and discuss the potential causes of previous failures. Additionally, this paper will analyze the therapeutic effects of chemotherapy, targeted therapy, and immunotherapy in clinical practice and look to provide individualized treatment strategies for patients with KRAS mutations in lung cancer.

## KRAS Biology

### KRAS Function

KRAS is a member of the RAS oncogene family and encodes a small membrane-bound GTPase that toggles between a bound state of active guanosine triphosphate (GTP) and a bound state of inactive guanosine diphosphate (GDP) (16–18). RAS proteins act like cellular switches that are controlled by stimuli, and when stimulated, in the GTP-bound form, these proteins activate diverse signaling pathways that regulate elemental cellular processes (19, 20).

**Figure 1:**
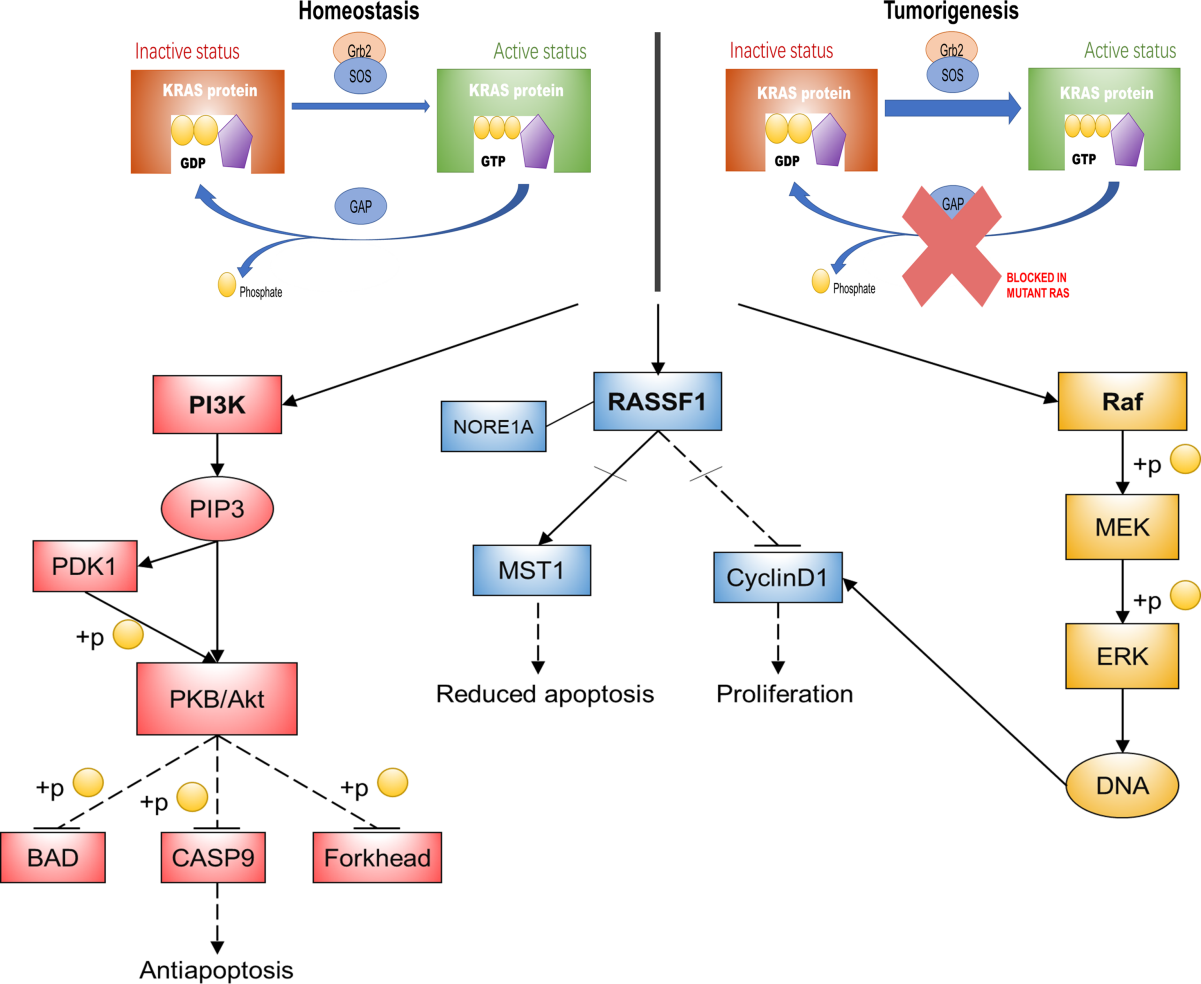
KRAS function and its main downstream pathways. This diagram is a summary of KRAS oncogenic mutations that impair its activity to hydrolyze GTP, thereby activating three major signaling pathways that mediate basic cellular processes. KRAS, kirsten rat sarcoma viral oncogene homolog; Gh2, growth hormone 2; SOS, Son of Sevenless; GTP, guanosine triphosphate; GDP, guanosine diphosphate; GAPs, GTPase-activating proteins; PI3K, phosphoinositide 3-kinase; Akt/PKB, protein kinase B; BAD, BCL-2/BCL-XL-associated death promoter; CASP9, Recombinant Caspase 9; PIP3, phosphatidyl inositol triphosphate; PDK1, 3-phosphoinositide dependent kinase-1; RASSF1, Ras association domain family 1; MST1, human macrophage stimulation 1; Raf, rat fibrosarcoma; MEK, mitogen-activated protein kinase kinase; ERK, extracellular regulated kinase; NORE1A, Ras-association domain family 5.

The activation of RAS signaling is strictly controlled by the regulatory factors that promote GDP–GTP exchange (guanine nucleotide exchange factors (GEFs)) or affect GTPase activity (GTPase-activating proteins (GAPs)). GEFs and GAPs are capable of binding to one or two pockets on RAS proteins, termed switch I and switch II regions, respectively. The former enhances the GDP release from RAS and stimulates its replacement by GTP, leading to RAS activation; the latter increases the inherent GTPase activity of RAS, leading to the rapid active–inactive transition of RAS state (21, 22). The main functional difference between mutant RAS oncoproteins and normal RAS oncoproteins is that mutant oncogene weakens the ability of RAS proteins to hydrolyze GTP (23–25). The RAS mutant oncoprotein is locked in a state of constitutive GTP-bound activity, leading to uncontrolled cell proliferation and survival (**Figure 1**). Therefore, RAS proteins are one of the mutant cellular proteins that were proven to be the driving force in human cancer. However, RAS proteins have not succumbed to any kind of targeted therapy and have even been known as “undruggable” for many years. This is because RAS proteins do not seem to provide suitable pockets that allow drug binding, except for their GDP/GTP binding sites. Unfortunately, what binds RAS proteins to these nucleotides is picomolar affinity, with very slow off-rates. In addition, GTPase signaling is mediated by protein–protein interactions (PPIs) involving extended and shallow surfaces. The tight binding and the high intracellular concentration of GTP make the identification of competitive nucleotide analogs seem almost impossible for a long time (26–28). Cysteine 12-modifying KRAS inhibitors that impair RAF binding and downstream signals (29); quinazoline-based compounds and guanosine mimetic inhibitors that suppress GTP loading of KRAS G12C and cell proliferation (30, 31); and allele-specific inhibitors that inhibit mutant KRAS-driven signaling by binding to the GDP-bound oncoprotein and preventing activation (32) are all recent discoveries that have started to shift the common perception that RAS proteins are undruggable.

### Downstream Effector Pathways

In addition to binding to GTP, RAS proteins must also establish a connection with the cell membrane to interact with GEF and other upstream regulators, such as EGFR, fibroblast growth factor receptor (FGFR), and human EGFR 2-4 (HER2-4/ERBB2-4). This happens so that extracellular signals can be transmitted to downstream signaling pathways (17, 18).

The biological effects of RAS depend on the signaling network it regulates. In this way, it is pivotal to understand not only the activation mode of RAS but also the mechanism of its downstream molecular effectors (33). There are more than ten reported RAS effectors implicated in multiple signaling cascades, including the canonical rat fibrosarcoma/mitogen-activated protein kinase/extracellular regulated kinase (Raf–MEK–ERK) pathway, a common overactivated pathway in cancer, which causes abnormal proliferation of cells by regulating the cell cycle. RAS also activates phosphoinositide 3-kinase/protein kinase B (PI3K–Akt/PKB) signaling, which plays a pivotal role in RAS protein-mediated antiapoptosis (34, 35). The RAS association domain family 1 (RASSF1) pathway is another RAS downstream effector pathway that is required for RAS-dependent apoptosis reduction and proliferation (36) (**Figure 1**).

In brief, RAS proteins play important roles in regulating cell proliferation, differentiation, and apoptosis by regulating signal transduction through different effectors that control diverse cellular functions. Constitutive activation of RAS oncoproteins initiates intracellular cascade reactions in the absence of extracellular signaling. This can lead to unlimited cell proliferation and aberrant cell survival. The deregulation of these cellular functions gives rise to hallmarks of cancer formation of various specificities (37).

### KRAS Mutations

**Figure 2:**
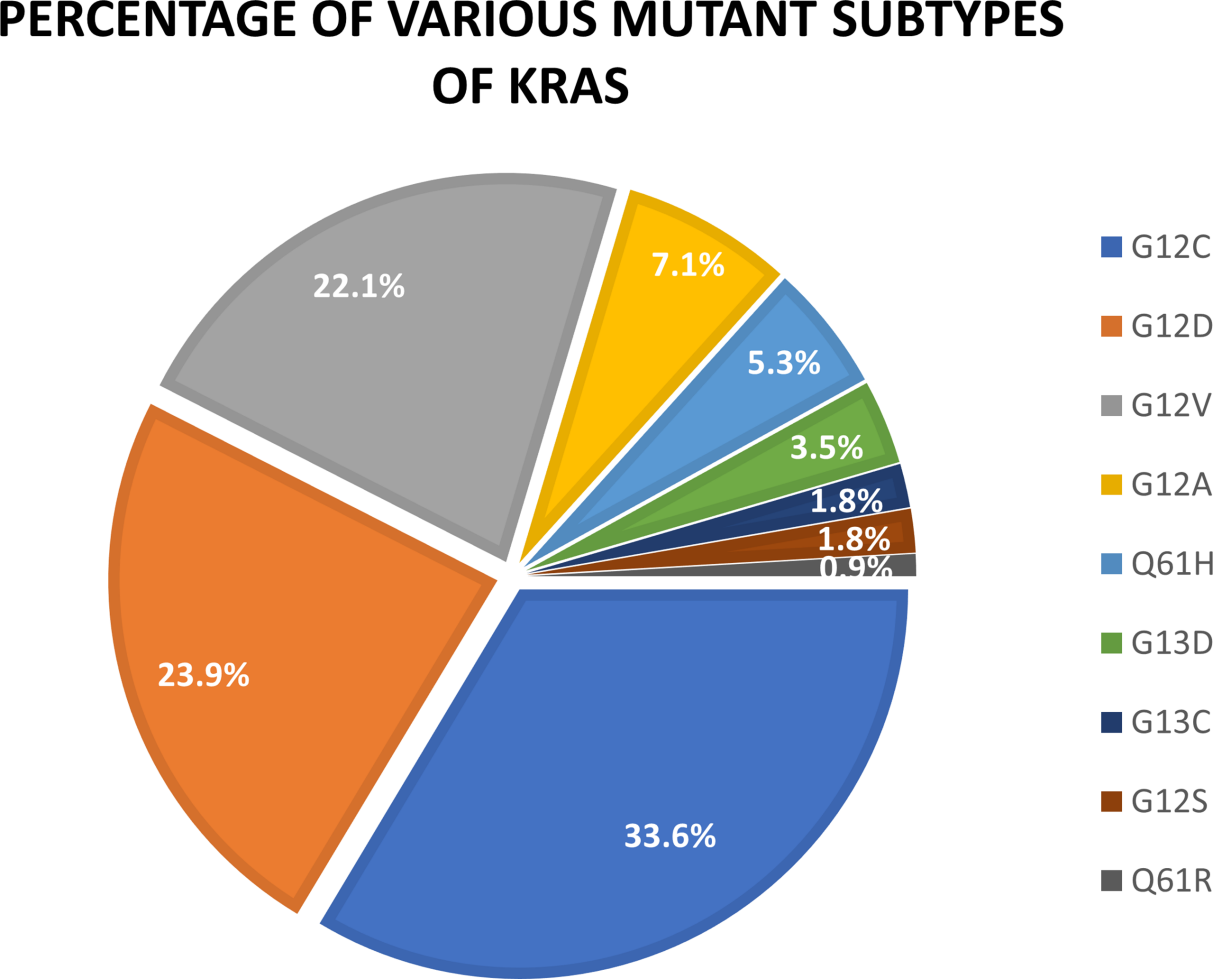
Percentage of various mutant subtypes of KRAS.

KRAS oncogenes are mainly mutations in exons 2, 3, and 4, which cause constitutive activation of the mitogen-activated protein kinase (MAPK) pathway. Approximately 90% of KRAS mutations occur at codon 12 (exon 2). This is especially noticeable in patients with NSCLC. The most common allele variants are G12C (GGT–TGT) and G12V (GGT–GTT), which are caused by classical smoking transformed from a G:C–T:A (38). The bioactive function of KRAS is related to the protein structure that depends on the bound state to GTP. Notably, KRAS mutations are heterogeneous and primarily involve substitutions in codons 12, 13, or 61 (39). In particular, G12 is situated on the p-ring and is involved in assisting nucleotide stability during activation, resulting in changes in intrinsic hydrolysis and gap-induced hydrolysis without changing the rate of nucleotide exchange (40). KRAS G12C and G12D are the most common types of mutations in lung cancer patients, accounting for 33.6% and 23.9% of total KRAS mutations, respectively. Other types of KRAS mutations are G12V (22.1%), G12A (7.1%), Q61H (5.3%), G13D (3.5%), Q13C (1.8%), G12S (1.8%), and G61R (0.9%) (41) (**Figure 2**).

**Figure 3:**
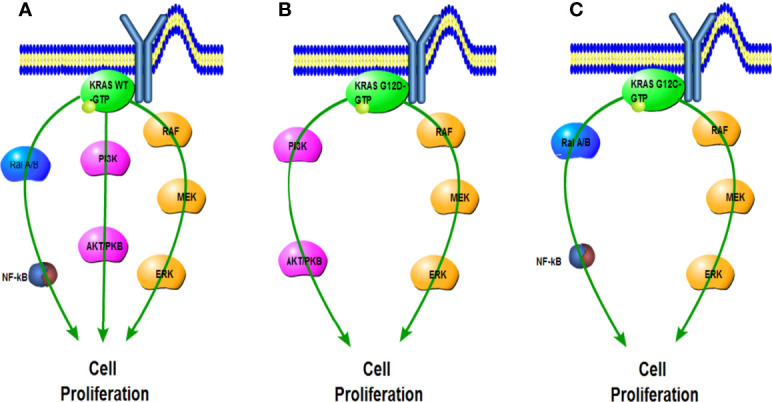
Impact of G12C and G12D mutations on KRAS downstream pathways compared with wild type. **(A)** Activated KRAS wild-type signals the Ral A/B, PI3K, and RAF pathways. **(B)** KRAS G12D preferentially activates the PI3K and RAF pathways. **(C)** KRAS G12C preferentially activates the Ral A/B and RAF pathways.

Specific KRAS mutations have unique biological characteristics. For example, although substitutions of KRAS G12, G13, and Q61 attenuate GTP hydrolysis capacity, other mutations such as KRAS A146T maintained hydrolysis levels similar to wild-type (WT) KRAS. The A146T substitution likely promotes KRAS-GTP formation in the form of increased nucleotide exchange, thereby reducing this isoform’s oncogenic capacity (42). Different types of KRAS mutations also cause differences in downstream signaling pathways. Basic experimental analysis revealed that lung cancer cell lines harboring KRAS G12C or KRAS G12V mutations exhibited increased Ras-related protein (RAL) A/B signaling but decreased PI3K/Akt signaling compared with other KRAS mutant isoforms or WT cell lines (43). Contrarily, cell lines containing KRAS G12D were more likely to activate the PI3K–Akt pathway (31, 44–47) (**Figure 3**).

## Current Approaches of KRAS-Mutant Non-Small Cell Lung Cancer and Their Efficacy in Different Subtypes

**Figure 4:**
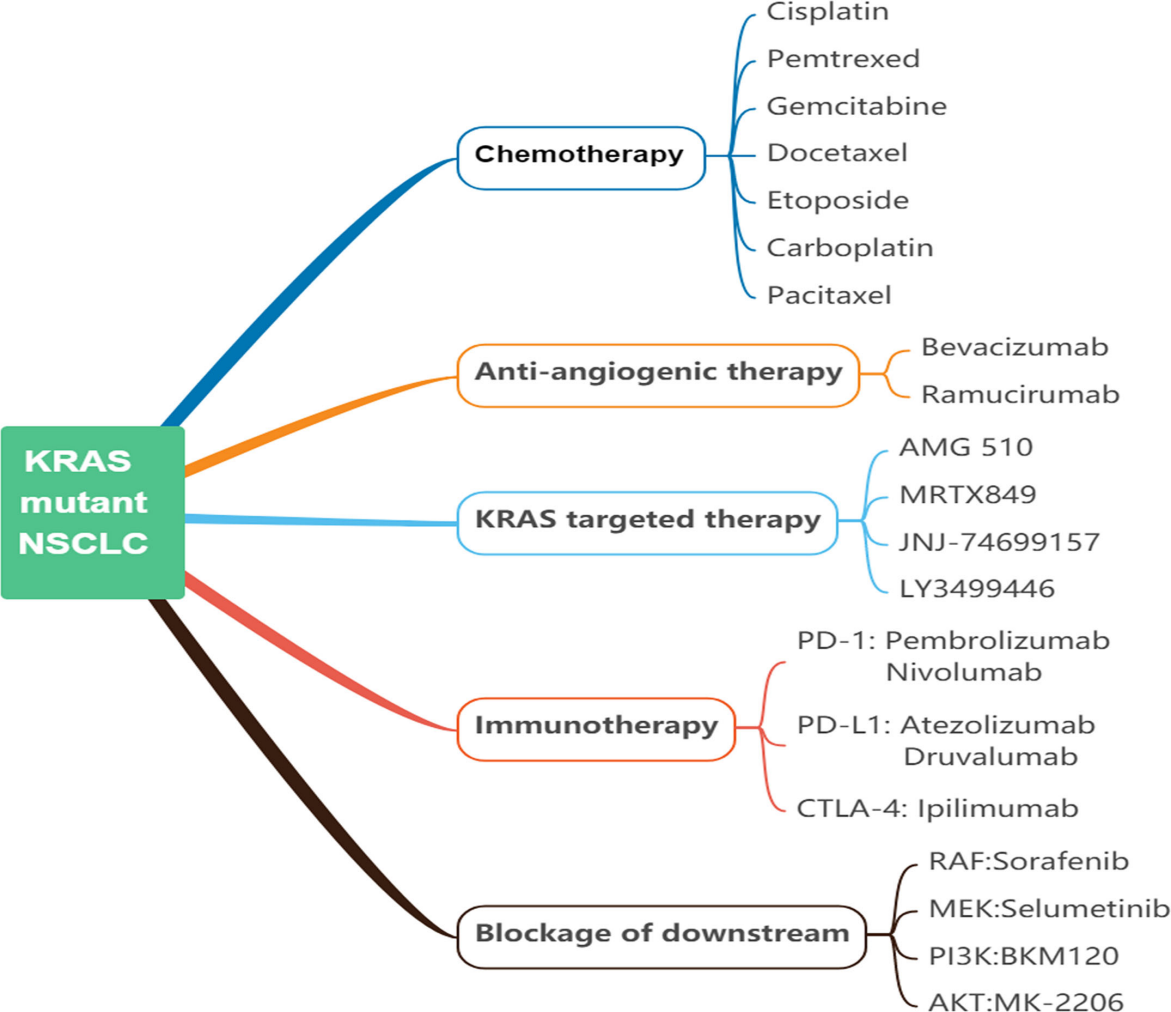
Current approaches of KRAS-mutant NSCLC. NSCLC, non-small cell lung cancer; KRAS, kirsten rat sarcoma viral oncogene homolog; PD-1, programmed cell death protein 1; PD-L1, programmed cell death-ligand 1; CTLA4, cytotoxic T-lymphocyte-associated protein 4; Raf, rat fibrosarcoma; MEK, mitogen-activated protein kinase kinase; PI3K, phosphoinositide 3-kinase; Akt/PKB, protein kinase B.

Although KRAS is one of the earliest oncogenic driver genes detected to date, no therapies have been found that effectively target KRAS mutations. Numerous therapeutic strategies have been developed including but not limited to chemotherapy, anti-angiogenic therapy, immunotherapy, blockage of downstream, and direct targeting of KRAS (18) (**Figure 4**). However, the vast majority of treatments have not been studied for individual KRAS mutation subtypes. In all subtypes of KRAS-mutant NSCLC, mutations occur primarily at codon 12 (>80%) and 13 (15%). Additionally, KRAS-G12C mutation accounts for approximately 39% of all KRAS mutants. Other frequently occurring mutations involve KRAS G12V (18%–21%) and KRAS G12D (17%–18%) variants (17). Further efforts are dedicated to elucidating the impact of different KRAS mutation subtypes in lung cancer patients on treatment efficacy. Different signaling and drug sensitivity patterns among these subtypes have been determined by preclinical studies, which suggested that differences may occur at the level of amino acid substitution (47, 48). Therefore, we reviewed the efficacy of the above treatments in different subtypes, aiming to provide ideas for personalized therapies of KRAS-mutant NSCLC (**Table 1**).

**Table 1:** Summary of clinical trials investigating the outcome of different KRAS mutation subtypes.

Study	Pts	KRAS status						Treatment	Endpoint	KRAS status					
Jia et al. (49)	170	WT	G12C	G12V	G12D	Rare		First-line chemotherapy		WT	G12C	G12V	G12D	Rare	
		59% (100)	14% (23)	11% (18)	5% (9)	11% (20)			ORR (%)	19.0	26.1	22.2	11.1	20.0	
									PFS (months)	6.4	4.4	2.9	7.0	4.7	
									DCR (%)	86	78.3	55.6	66.7	60	
Cserepes et al. (50)	494	WT	G12C	G12V	G12D	Rare	COD13 MUT	First-line chemotherapy		WT	G12C	G12V	G12D	Rare	COD13 MUT
		68% (338)	12% (61)	6% (29)	6% (27)	4% (19)	4% (20)		PFS (days)	211	191	233	150	198	157
									OS (days)	479	561	470	325	559	330
Sun et al. (51)	304	WT	G12C	G12V	G12D	Rare		First-line chemotherapy		WT	G12C	G12V	G12D	Rare	
		87% (265)	3% (9)	3% (10)	4% (13)	3% (7)			OS (months)	15	7.7	9.6	8.1	5.5	
Ghimessy et al. (52)		WT	G12C	G12V	G12D	Rare		BEV/CHT		WT	G12/13X	G12D			
	213	61% (130)	16% (35)	10% (20)	9% (19)	4% (9)			PFS (months)	11.70	8.27	3.70			
									OS (months)	21.0	16.1	7.2			
		G12C						Sotorasib		G12C					
Skoulidis et al. (53)	124	124							ORR (%)	37.1					
									PFS (months)	6.8					
Jeanson et al. (54)		G12A	G12C	G12D	G12V	G13C		ICIs		G12A	G12C	G12D	G12V	G13C	
	144	10% (15)	48% (69)	17% (25)	17% (24)	8% (11)			ORR (%)	13.3	18.5	20.0	18.2	18.2	
									PFS (months)	2.66	3.09	3.91	2.69	4.60	
Jänne et al. (55)		G12C/V	Non-G12C/V					Selumetinib + docetaxel		G12C/V	Non-G12C/V				
	83	57% (47)	43% (36)						PFS (months)	5.7	4.9				
									OS (months)	9.6	8.6				
								Placebo + docetaxel	PFS (months)	1.4	2.6				
									OS (months)	4.4	7.1				

KRAS, kirsten rat sarcoma viral oncogene homolog; Pts, patients; ORR, objective response rate; PFS, progression-free survival; DCR, disease control rate; OS, overall survival; BEV, bevacizumab; CHT, chemotherapy; ICIs, immune checkpoint inhibitors.

### Status of Chemotherapy in Patients With Different KRAS Mutation Subtypes of Non-Small Cell Lung Cancer

In recent advances in the treatment of NSCLC, most patients with the advanced-stage disease are still treated with platinum-based chemotherapy regimens. The predictive value of KRAS mutations in NSCLC was investigated in patients receiving definitive chemotherapy (56), postoperative radiotherapy adjuvant chemotherapy (57), or the phase III TRIBUTE trial comparing first-line carboplatin/paclitaxel with erlotinib or placebo for advanced NSCLC (58). In the above settings, KRAS mutation has not been found to be a predictor of response rate, progression-free survival (PFS), or overall survival (OS) in patients with lung cancer. The JBR10 trial showed a significant positive effect with chemotherapy in only KRAS WT. Yet the difference was not shown to be statistically significant (p = 0.29) (59). In addition, an Asian cohort study analyzed the prognosis of lung cancer patients who received different chemotherapy regimens according to KRAS mutation status. OS was markedly worse in KRAS mutant patients treated with pemetrexed or gemcitabine (p = 0.12). Meanwhile, among KRAS mutated lung cancer patients, OS was longer and statistically significant for adenocarcinoma patients compared with squamous carcinoma patients (22.7 vs. 11.5 months; p = 0.051) (51). It is worth noting that clinical studies show that PFS and OS are remarkably shortened in patients with KRAS codon 13 mutation, which indicates that the KRAS codon 13 mutation has a potential negative impact on chemotherapy (60). Additionally, analysis of clinical data from platinum-based chemotherapy according to KRAS mutation status demonstrated that patients with mutations at codon 13 experienced a shorter PFS and OS compared when compared with patients with mutations at codon 12 (61). According to a retrospective study that involved 2,183 Chinese cases that exhibited KRAS mutations, patients with KRAS G12V mutations seemed to have a poorer response to chemotherapy than others. In this study, a shorter and statistically significant PFS was observed when the G12V mutant was compared with WT patients (2.9 vs. 6.4 months, respectively; p = 0.001). Patients with KRAS G12V mutations were less sensitive to chemotherapy and had worse PFS than non-KRAS G12V mutated patients (median PFS (mPFS), 2.9 vs. 4.7 months; p = 0.046). There was no difference in PFS for other KRAS subtypes that were compared. In addition, PFS may be shorter in patients with KRAS mutated adenocarcinoma histology (4.3 vs. 6.7 months; p = 0.051). However, KRAS WT patients had a significantly higher disease control rate (DCR) to platinum-based chemotherapy (86.0% vs. 65.7%, p = 0.002). Although G12V had the lowest DCR of 55.6%, the response profile to platinum-based chemotherapy did not appear to be statistically significant between mutational subtypes (p < 0.05) (49). However, in a recent study, patients with KRAS G12V mutant lung adenocarcinoma (LUAD) not only tended to have a better response to platinum-based chemotherapy (p = 0.077) but also, although there was no significant difference, were more likely to have longer PFS than patients with other codon 12 mutations (p = 0.145) (50). However, these differences were not statistically significant. In these results, it can be found that the KRAS 13 codon is less sensitive to chemotherapy than the KRAS 12 codon. G12V showed poor efficacy in first-line chemotherapy, but it showed strong sensitivity to platinum-based chemotherapy. However, the KRAS mutant is less sensitive to chemotherapy than the KRAS WT.

Therefore, we propose a clinically significant hypothesis, namely, that the different types of KRAS mutations can produce different reactions to chemotherapy. This was seen in a study of cell lines with KRAS mutations by Garassino et al. In comparison with the WT clones, the G12C mutation was associated with a reduced response to cisplatin but increased sensitivity to taxol and pemetrexed, whereas G12V mutation showed a strong sensitivity to cisplatin but less sensitivity to pemetrexed. For cell lines with G12D mutations, taxol had minimal effects, but sorafenib had sound results (48).

### Status of Anti-Angiogenic Therapy in Patients With Different KRAS Mutation Subtypes of Non-Small Cell Lung Cancer

In addition to initiating tumor formation by stimulating proliferation, oncogenic RAS ensures tumor progression by promoting tumor angiogenesis (62, 63). Different downstream pathways of oncogenic RAS are ultimately involved in promoting tumor angiogenesis through the upregulation of vascular endothelial growth factor (VEGF) and CXC chemokine interleukin-8 (IL-8) (62, 64–67). Angiogenesis inhibition is one of the most important strategies against solid tumors. Cutting off the blood supply to a tumor micro area leads to a lack of oxygen to the solid tumor; this results in extensive tissue necrosis within the tumor organization. The difference between normal and tumor tissue angiogenesis activation makes the process of antitumor drug discovery an attractive strategy target (68). Over the past decades, the VEGF signaling pathway has been identified as a central axis in the process of tumor angiogenesis. The advent of recombinant antibody technology has facilitated the development of bevacizumab (BEV), a humanized antibody that targets VEGF and is the current leading clinical treatment to inhibit tumor angiogenesis (69).

However, although it has been proved that VEGF plays an indispensable role in tumor angiogenesis mediated by RAS, seldom do we have studies involving the relationship between KRAS mutations and antiangiogenic therapy efficacy (70–72). A phase II trial assessing the efficacy of BEV in chemotherapy found that all KRAS mutated patients had no pathological response to neoadjuvant BEV combined with chemotherapy, whereas 35% of patients with WT KRAS showed a significant pathological response (73). In a recent clinical retrospective study by Ghimessy et al., patients with KRAS mutations, and especially patients with KRAS G12D mutant lung cancer, had a significantly shorter OS than those with KRAS WT or other KRAS mutant tumors (p = 0.0223 and p = 0.0144, respectively). At the same time, the KRAS WT or all other codon 12/13 (G12/13x) KRAS mutations other than KRAS G12D mutation had significant adverse effects on PFS (p = 0.0032). Thus, G12D mutations may define a subset of KRAS types for which LUAD patients with such mutations are not eligible for treatments with BEV-based antiangiogenic drugs (52).

### Status of KRAS Targeted Therapy in Patients With Different KRAS Mutation Subtypes of Non-Small Cell Lung Cancer

Although KRAS was discovered decades ago, none of the studies targeting KRAS therapy have achieved significant results until recent years. Several studies have shown that specific mutant KRAS may cause differential sensitivity to EGFR tyrosine kinase inhibitors (EGFR-TKIs). One such study demonstrated that patients with the KRAS codon 13 mutation experienced worse outcomes when compared with patients with KRAS codon 12 mutations and KRAS WT patients (p < 0.0001 and p = 0.01 for PFS and OS, respectively) (74). Another study proved the potential OS benefit of EGFR-TKIs in patients with KRAS G12D/G12S mutations (HR = 0.49, p = 0.05). It was also observed that EGFR-TKIs tended to reduce survival in patients with G12C/G12V mutations (HR = 1.41, p = 0.07), which was more significant in the adenocarcinoma subgroup (HR = 1.73, p = 0.01), while the harmful effects of G12V mutation alone were more prominent (HR = 1.96, p = 0.04) (75). Contrarily, Fiala et al. reported that EGFR-TKIs improved PFS in patients with non-G12C KRAS mutant tumors when compared with the G12C group (76). However, the poor outcomes of EGFR WT/KRAS-mutant NSCLC patients indicate that the KRAS mutation is neither prognostic nor predictive of benefit from EGFR-TKIs (77). Recent advancement in RAS targeted drugs is the development of allele-specific inhibitors. The locations of KRAS oncogenic mutations are mainly clustered at several hotspot residues, especially in G12 (78). KRAS G12C mutants have cysteine residues that have been used to design covalent inhibitors with preclinical activity recently, which makes the inability to drug KRAS a thing of the past (29, 32, 79). Mutation-selective KRAS inhibitors utilize reactivity and the nucleophilic cysteine at No. 12. Thus, modified by disulfide bonds, these covalent conjugates can be incorporated into allosteric isomers and allosterically inhibit KRAS oncoprotein activity, or bind to the orthosteric substrate site and compete with GDP/GTP to inhibit protein activation. Ostrem et al. found compounds 6 and 12 and identified their corresponding new allosteric site switch II pocket (S-IIP), which opened the way for the development of allosteric KRAS G12C covalent inhibitors (29).

AMG510 is a small-molecule compound that irreversibly and specifically binds to G12C and functions to lock KRAS in an inactive state with GDP. This covalent inhibitor slowly converts the KRAS active state to the KRAS-GDP state with a 30-min half-life. In a recently concluded phase II clinical trial (NCT 03600883) in which 124 patients were evaluated, 37.1% patients with NSCLC had a confirmed objective response (4 had a complete response and 42 had a partial response; 95% CI, 28.6 to 46.2), and 80.6% had a disease control response (95% CI, 72.6 to 87.2); the mPFS was 6.8 months (95% CI, 5.1 to 8.2) (53). Another ongoing phase I/II trial targeting KRAS G12C (NCT 03785249) considers MRTX849, a similar small-molecule direct inhibitor with a half-life of 20 h, irreversibly binds to cysteine 12 in the switch II pocket induced by KRAS G12C and locks the KRAS protein in an inactive GDP bound state, resulting in the inhibition of the RAS/MAPK signaling pathway (80). A phase I trial (NCT04006301) conducted by Janssen evaluating JNJ-74699157 has just begun recruitment. The drug is an investigational, orally available small molecule that is designed to potently and selectively inhibit KRAS G12C (81). Eli Lilly drug LY3499446, a new compound under development as KRAS G12C inhibitors (NCT #04165031), will be evaluated as a single agent or in combination with other agents such as abemaciclib, cetuximab, and erlotinib in advanced solid tumors including NSCLC (81). We can conclude that NSCLC patients with KRAS G12D/G12V/G13C mutations are better candidates for immunotherapy than patients with KRAS G12A/G12C mutations.

### Status of Immunotherapy in Patients With Different KRAS Mutation Subtypes of Non-Small Cell Lung Cancer

It is widely indicated that the degree of programmed cell death protein 1 (PD-1) expression is tightly correlated with the KRAS subtype status, and KRAS mutation is, to some extent, considered a possible biomarker for immune checkpoint inhibitors (ICIs) (82). Furthermore, clinical benefit from the application of PD-1 inhibitors in patients with KRAS mutations was reported in a comprehensive analysis (83). Increased expression of PD-1 has been affirmed in KRAS mutant cells, accompanied by the demonstration that ERK activation mediates upregulation of programmed cell death-ligand 1 (PD-L1) through KRAS mutations (84). A study based on the intrinsic link between the degree of PD-L1 expression on tumor cells and the type of KRAS mutation found that, as a PD-1 inhibitor, pembrolizumab, or an ERK inhibitor can restore the body’s antitumor immunity and prevent apoptosis of CD3+ T cells by preventing the immune escape of tumor cells (85). At the same time, a large number of studies have confirmed that PD-L1 expression has a close relationship with circulating tumor cells (CTCs). Reduced CTC numbers are strongly associated with a good response to immunotherapy and longer OS and PFS (86, 87). Additionally, other available data indicate that high CTC values before treatment are associated with an increased risk of patient death and progression (88). The results of Wang et al. showed that CTCs can also be used to detect the dynamic changes of PD-L1 during radiotherapy in lung cancer patients (89). Nicolazzo et al. summarized that stage IV patients with NSCLC who received ICI nivolumab therapy could have their resistance to immunotherapy measures through the persistence of PD-L1-positive CTCs (90). In addition, there is evidence for variability in the biological behavior of different KRAS mutation subtypes due to the high heterogeneity in the presentation of KRAS mutations. Therefore, attention needs to be paid to heterogeneity in the efficacy of immunosuppressive agents in lung cancer patients with KRAS mutations when immunotherapy is administered. Meanwhile, differences in the tumor microenvironment (TME) of different lung cancer patients affect the efficacy of immunotherapy. There is evidence that the above TME differences can affect the sensitivity of lung cancer patients to immunotherapy. Some of these differences are the status of neutrophils, the number of NK cell counts, the activity of dendritic cells (DCs), the expression of PD-L1 on macrophages, Foxp3+ Ti/S ratio, and CD8+ Ti/S ratio, and chromosomal stability (91–96). It should also be mentioned that gut microbiota can shape TME by modulating the immune and hormonal factors throughout the host (97, 98). The metabolites of the gut microbiota also have implications for the TME and tumor immunosuppressive therapy (99–101). Modulation of the gut microbiota has been reported to enhance the effects of cancer immunotherapy (101). Therefore, when administering immunotherapy to patients with KRAS mutated lung cancer, attention needs to be paid to the differences in the TME of patients with lung cancer while paying attention to the heterogeneity in the efficacy of immunosuppressive agents. This is all with respect to the expectation of achieving the individualization of immunotherapy for patients with lung cancer.

In a retrospective study, Jeanson et al. analyzed the extent of PD-1 expression in 128 patients with advanced NSCLC (all histological subgroups, predominantly LUAD) treated with ICIs (anti-programmed death 1, anti-PD-L1, or anti-cytotoxic T-lymphocyte-associated protein 4 antibodies). Although no significant differences were observed when comparing the efficacy and toxicity of ICIs between different subtypes of KRAS mutations, there were statistically significant differences in PD-L1 expression: a higher proportion of patients with KRAS G12D, G12V, or G13C mutations had PD-L1 positive tumors, and a higher proportion of PD-L1-negative tumors had G12A and G12C mutations. Interestingly, KRAS-mutant NSCLC was investigated according to the degree of PD-L1 expression; they found that a better objective response rate (ORR) and longer PFS were observed for PD-L1-positive tumors. Meanwhile, in patients with KRAS G12A and G12V mutant cancers, the degree of PD-L1 expression was similar to the ORR and PFS in patients treated with ICIs (54). We may conclude that NSCLC patients with KRAS G12D/G12V/G13C mutations are better candidates for immunotherapy, whereas patients with KRAS G12A/G12C mutations are not.

### Downstream Pathway Inhibitors Vary Between Patients With Different KRAS Mutation Subtypes of Non-Small Cell Lung Cancer

The KRAS signaling pathway is highly complex and dynamically changing, and the downstream pathways involve multiple effectors. The representative ones are the Raf–MEK–ERK and PI3K–Akt signaling networks (102, 103). Due to the slightly different biochemical characteristics of each allele, the downstream pathways involved may vary in quantity and quality (104). The best existing example is the varying sensitivity of CRC cell lines expressing different KRAS alleles to MEK1/2 inhibition. Cell lines expressing A146T were sensitive to a single MEK1/2 inhibitor, but not to other KRAS-activated mutations (105). Also, the effects of different KRAS mutation subtypes on downstream signaling pathways such as PI3K may result in differential response to therapy (47). Therefore, it is important to understand whether the metabolic levels of cells with different KRAS mutation statuses are affected by these inhibitors. This will help to inform new combination regimens that have the potential to form targeted therapies for WT and mutant cancer cells to help patients receive tailored treatment.

Caiola et al. studied KRAS WT and G12C mutated NSCLC clones to determine the response of both to PI3K–Akt inhibitors (BEZ235 and BKM120). Metabolomic analysis revealed that although the final effects of both mutation types on cell growth, cell cycle distribution, and caspase activation were similar, glutamine metabolism in KRAS G12C and serine metabolism in KRAS WT were impaired after PI3K signaling pathway blockade by inhibitors. PI3K inhibitors cause autophagy in KRAS WT, but not KRAS G12C. At the same time, there was significantly reduced KRAS G12C ammonia production, possibly as a result of impaired glutamine metabolism (106). A randomized phase II trial of selumetinib plus docetaxel in KRAS mutant advanced NSCLC suggested the impact of KRAS codon subtypes. Patients with KRAS G12V mutation had longer PFS and ORR than other subtypes (p = 0.24 and p = 0.189, respectively), while KRAS G12C mutation may have longer OS than other mutation types (p = 0.48). Further analysis at week 6 suggested tumors harboring KRAS G12V may have had a better response: G12V (n = 9) for 62%; reduction across all codons (n = 81) for 18% (55).

## Impact of KRAS Concurrent Pathogenic Mutations on Outcomes of Therapy

KRAS-mutant NSCLC has been proven to be a genetically heterogeneous disease. In addition to having different types of point mutations, it is often associated with other co-mutations in lung cancer, which has been reported in various papers in recent years (107, 108). Approximately 50% of NSCLC with KRAS mutations have additional co-accompanied mutations that are critical in tumorigenesis, such as TP53, STK11/LKB1, KEAP1, and SAMARCA4—which are the most commonly reported mutations (109). We summarized the clinical trials that are investigating the outcomes of different KRAS co-mutations below, in order to provide references for the personalized treatments of relevant patients (**Table 2**). Each of these co-mutational partners is a key contributor to Ras signaling and the TME in lung tumor cells and has resulted in more prominent molecular and clinical heterogeneity of KRAS-driven NSCLC (38).

**Table 2:** Summary of clinical trials investigating the outcomes of different KRAS co-mutations.

Study	Pts	KRAS status			Treatment	Endpoint	KRAS status		
Skoulidis et al. (110)		K-only	KP	KL	ICIs		K-only	KP	KL
	174	37% (64)	32% (56)	31% (54)		ORR (%)	28.6	35.7	7.4
						PFS (months)	2.7	3.0	1.8
						OS (months)	16.1	16.0	6.4
Skoulidis et al. (53)		KRAS MUT	KRAS-KEAP1 MUT		Sotorasib		KRAS MUT	KRAS-KEAP1 MUT	
	104	81% (84)	19% (20)			ORR (%)	44	20	
Alessi et al. (111)		K-only	KS		ICIs		KS	K-only	
	176	90% (159)	10% (17)			ORR (%)	0	22.0	
						PFS (months)	1.4	4.1	
						OS (months)	3.0	15.1	
Liu et al. (112)		K-only	KP	KS	Non-immunotherapy		K-only	KP	KS
	155	61% (94)	33% (52)	6% (9)		DFS (months)	18.0	16.31	10.97
						OS (months)	20.11	18.48	15.37
					ICIs				
	77	56% (43)	32% (25)	12% (9)		PFS (months)	2.77	4.63	1.73

KRAS, kirsten rat sarcoma viral oncogene homolog; Pts, patients; KL, KRAS-STK11/LKB1 co-mutant; KP, KRAS-TP53 co-mutant; ORR, objective response rate; PFS, progression-free survival; OS, overall survival; MUT, mutation; KEAP1, Kelch-like ECH-associated protein 1; ICIs, immune checkpoint inhibitors; KS, KRAS-SMARCA4 co-mutant; DFS, disease-free survival.

### KRAS Co-Mutated With TP53 and STK11/LKB1

KRAS mutations in NSCLC patients frequently occur together with mutations in tumor protein 53 (TP53) and serine–threonine kinase 11/liver kinase B1 (STK11/LKB1). Genomic alterations co-occurred in the TP53 and STK11/LKB1 tumor suppressor genes, which define the unique biology, therapeutic sensitivities, and immune conditions of different subgroups of NSCLC with KRAS mutations (113). STK11 / LKB1 encodes a serine threonine kinase, which plays a role in cell metabolism ,energy homeostasis, growth and polarity regulation through the phosphorylation of adenosine monophosphate activated protein kinase (AMPK) and 12 AMPK related kinases. (114). Inactivation of STK11 (or its protein product LKB1) through mutational or non-mutational mechanisms has been associated with an inert or “cold” TME. It leads to the accumulation of neutrophils with T cell-suppressive effects, accompanied by a corresponding increase in the expression of T-cell exhaustion markers and tumor-promoting cytokines. In human tumors and genetically engineered mouse models, the density of invasive cytotoxic CD8+ T lymphocytes was decreased, along with the reduced expression of PD-L1 (113, 115, 116). In contrast, extensive infiltration of cytotoxic CD8+ Th1 tumor-infiltrating lymphocytes (TILs), as well as high expression of interferon (IFN)-dependent genes and IFN-induced PD-L1, was predominantly observed in KRAS-TP53 co-mutated tumors (110).

In the study of Skoulidis et al., LUAD patients were divided into three groups according to whether TP53 or STK11/LKB1 gene mutations occurred. The majority of KRAS-STK11/LKB1 co-mutated (KL) tumors were shown to be significantly more resistant to PD-1 inhibitors, with lower response rates observed for this subtype in three independent databases [9.1% for MDACC (MD Anderson Cancer Center), 9.1% for MSKCC (Memorial Sloan Kettering Cancer Center), and 4.8% for DFCI/MGH (Dana-Farber Cancer Institute/Massachusetts General Hospital)]. On the other hand, the KRAS-TP53 co-mutant (KP) group showed greater sensitivity to PD-1 inhibitors. When it comes to PFS, the KL group showed significantly shorter PFS than either K-only (hazard ratio (HR) 1.98, 95% CI, 1.33 to 2.94; p < 0.001) or KP (HR 1.77, 95% CI, 1.16 to 2.69; p = 0.0072) groups in pairwise comparisons, while the latter two groups had similar PFS. Meanwhile, this significant difference in OS was also observed among the three subgroups in the SU2C cohort and was statistically significant (p = 0.0045). Median OS was 6.4 months in KL compared with 16.0 months in KP and 16.1 months in K-only LUAD (110). In the study by La Fleur et al., worse OS was observed for LUAD patients with a mutation in either TP53 or STK11/LKB1. In the LUAD KRAS mutation group, poor survival appeared to be related to TP53 or STK11/LKB1 co-mutations instead of a single KRAS aberration. This result was also found in the open data analysis of cBioPortal (117).

Co-mutations in KRAS and TP53 suggest that in lung cancer, tumors carrying these mutations may be more sensitive to immune checkpoint suppression (83). Conversely, tumors with both KRAS and STK11 mutations may be associated with an immunosuppressive microenvironment (110, 118). In addition, in the presence of oncogenic KRAS mutations, STK11/LKB1 deficiency promoted the synthesis of interleukin-6 (IL-6), which predominantly recruited large numbers of neutrophils but suppressed T-cell infiltration, and it had higher criteria for markers of T-cell exhaustion (mainly PD-1). Moreover, PD-L1 expression was also suppressed in cancer cells, indicating that STK11-deficient KRAS mutations lead to anti-PD-1/PD-L1 resistance in cancer cells (110, 115).

In summary, KRAS-TP53 co-mutant NSCLC patients are more suitable for treatment with ICIs, while those with both KRAS and STK11/LKB1 mutations demonstrated resistance.

### KRAS Co-Mutated With KEAP1

Kelch-like ECH-associated protein 1 (KEAP1), a principal repressor of nuclear factor erythroid 2-like 2 (NFE2L2; hereafter NRF2), functions primarily as a transcriptional regulator during the cellular oxidative stress response and is one of the most frequent co-mutations in KRAS mutated tumors that co-occur with genomic changes that affect tumor biology and response to systemic therapy (92, 104, 105, 119). According to a pan-cancer analysis, the amount of KEAP1 mutations in 40,167 patients with distinct cancer types was 2.7%; patients with NSCLC had the highest levels of KEAP1 mutations (15.8%) (120). Nearly 20% of KRAS mutant lung cancers harbor concurrent loss of function (LOF) mutations in KEAP1 (121–123).

The results demonstrate that KEAP1 mutations activate the NRF2 antioxidant program and promote LUAD progression in concert with mutant KRAS (124), demonstrating that cancer cells can overcome oxidative stress barriers during tumorigenesis (125–131). The metabolic requirement for glutaminolysis may also similarly manifest as a therapeutic vulnerability in other cancers with genetic (132–137), epigenetic (138–140), or post transcriptional (141) alterations in the KEPA1/NRF2 signaling pathway, a hypothesis that illustrates the importance of kinase-targeted therapeutic strategies for KRAS-KEAP1 mutant lung cancer (142). Furthermore, in KRAS-mutant LUAD, tumors with LKB1 loss are highly enriched for concurrent KEAP1 mutations, which activate the KEAP1/NRF2 pathway. A recent study investigated the biological consequences of these co-occurring alterations and explored whether they conferred specific therapeutic vulnerabilities. The results collectively found that in kallikrein-related peptidases (KLK) tumor cells, activation of the KEAP1/NRF2 pathway limits metabolic flexibility and promotes glutamine-addictive metabolism to maintain the tricarboxylic acid (TCA) cycle in addition to redox homeostasis, rendering these tumor cells selectively vulnerable to glutaminase inhibitors (143).

In an exploratory analysis by Skoulidis et al., the activity of sotorasib was observed across a spectrum of prevalent co-occurring mutations, including STK11 and KEAP1, both of which are related to inferior treatment outcomes and a poor prognosis in patients with NSCLC (110, 117, 120, 144–147). Among the 104 patients (KRAS-mutant NSCLC is mainly adenocarcinoma, accounting for 95.2%) who were assessed for co-occurring genomic alterations, efficacy was seen in the subgroups with mutated STK11, KEAP1, or TP53. After total genomic changes were assessed in 104 patients, efficacy was significantly improved in the STK11, KEAP1, or TP53 mutated subgroups. Fifty percent (95% CI, 28 to 72) of patients in the STK11 mutant subgroup and WT KEAP1 subgroup responded, and 39% (95% CI, 30 to 49) of evaluable patients responded. Among patients with KEAP1 mutations, 23% of patients in the STK11 and KEAP1 subgroups (95% CI, 5 to 54) responded, compared with 14% of patients in the WT STK11 and KEAP1 subgroups (95% CI, 0 to 58) (53). In total, KEAP1 co-mutation is an adverse factor for NSCLC patients with KRAS mutations who receive sotorasib therapy.

### KRAS Co-Mutated With SMARCA4

The SWItch/Sucrose Non-Fermentable (SWI/SNF) chromatin remodeling complexes control DNA accessibility to transcriptional factors and regulate transcriptional programming (148, 149). The genes encoding SWI/SNF complex subunits are among the most highly mutated in cancer. Among various kinds of cancer, SWI/SNF multi-subunit protein complex composition of genomic changes has taken place. It is estimated that at least 20% of malignancies have SWI/SNF complex subunit mutations (150, 151). SWI/SNF related, matrix associated, actin-dependent regulator of chromatin, subfamily A, member 4 (SMARCA4) encodes brahma-related gene1 (BRG1), one of two mutually exclusive ATPase subunits of the SWI/SNF complex. Mutations in the SMARCA4 gene are found in a variety of cancers and tended to co-occur with KRAS mutations frequently (10%) (122, 152–155). Studies have shown that inactivation of SMARCA4 promotes the invasion of NSCLC by altering chromatin organization (156), while decreased expression of SMARCA4 results in a poor prognosis of lung cancer (157–159).

Lissanu et al. showed that SMARCA4 through synergies with lack of p53 and KRAS activation plays a role of tumor suppressor, and these SMARCA4 mutations in the tumor were highly sensitive to the inhibition of oxidative phosphorylation (160). Another study indicated that decreased expression of SMARCA4 resulted in a poor prognosis of lung cancer. Besides, the presence of SMARCA4 co-mutations in KRAS mutated NSCLC patients was found to contribute to poor immunotherapy outcomes (157). Besides, the presence of SMARCA4 co-mutations in KRAS mutated NSCLC patients was found to contribute to poor immunotherapy outcomes (111, 161). In the study of Alessi et al., compared with K-only subgroup, ORR (22% vs. 0%, p = 0.03), mPFS (4.1 vs. 1.4 months, HR = 0.25, 95% CI, 0.14 to 0.42, p < 0.001) and median OS (15.1 vs. 3.0 months, HR = 0.29, 95% CI, 0.17 to 0.50, p < 0.001) in KRAS-SMARCA4 co-mutant (KS) subgroup were significantly shortened (149). To make the conclusions more comprehensive, the analysis by Liu et al. concluded that genomic changes in SMARCA4 are one of the reasons for the poor prognosis of KRAS mutant LUAD patients regardless of whether they received non-immunotherapy or immunotherapy. Among patients receiving non-immunotherapy, the KS subgroup had a significantly shorter DFS than the KP (HR 4.47, 95% CI, 1.52 to 13.22, p = 0.003) and K-only (HR 2.43, 95% CI, 1.46 to 4.05, p = 1.2E−4) two subgroups. A retrospective review of LUAD patients treated with immunotherapy yielded that the KS co-mutated group had shorter PFS than the other subtypes in various subgroup analyses (HR = 2.82, 95% CI, 1.17 to 6.81, p = 0.016) (112).

NSCLC patients with concurrent KRAS and SMARCA4 mutations require another targeted therapeutic strategy. Cisplatin-based chemotherapy was shown to be beneficial to patients with NSCLC with low SMARCA4 expression in a clinical study (157). CDK4 inhibitors including palbociclib may also be a potential alternative (162). In addition, a recent study showed that SMARCA4-deficient lung cells and xenograft tumors suppressed oxidative phosphorylation evidently (160). All observations suggest that therapeutic strategies are encouraged, but further clinical trials are needed.

## Conclusion

**Figure 5:**
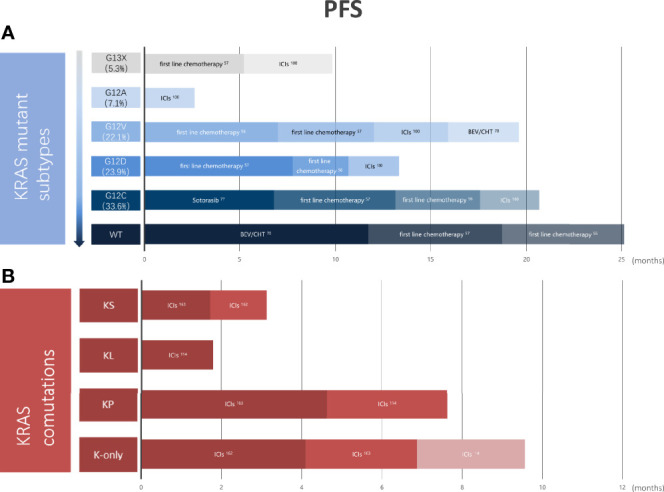
Outcomes vary in primary treatments in different KRAS mutation subtypes or different KRAS co-mutations. **(A)** Pooled PFS of NSCLC patients with KRAS wild-type and different KRAS mutations from clinical trials. **(B)** Pooled PFS of K-only and KRAS co-mutated patients with NSCLC from clinical trials. KRAS, kirten rat sarcoma viral oncogene homolog; COD, codon; WT, wild type; ICIs, immune checkpoint inhibitors; BEV, bevacizumab; CHT, chemotherapy; KS, KRAS-SAMARCA4 co-mutant; KL, KRAS-STK11/LKB1 co-mutant; KP, KRAS-KEAP1 co-mutant; PFS, progression-free survival; NSCLC, non-small cell lung cancer.

As of today, differences in the effectiveness of chemotherapy, antiangiogenic therapy, targeted therapy, or immunotherapy among lung cancer patients with different KRAS mutant subtypes are not known. However, the above research results show that G12C/V is effective for platinum-based chemotherapy, while G12D is more sensitive to first-line chemotherapy. EGFR inhibition has a poor effect on KRAS mutation, but codon 12 mutations are more sensitive than codon 13 mutations. Meanwhile, patients with KRAS G12C lung cancer are likely to find success with covalent inhibitors such as AMG 510 and MRTX849, an anti-endogenous protein degradation molecule. Codon 13 mutations are more sensitive to ICIS than codon 12 mutations. KRAS co-mutated with STK11/LKB1 is insensitive to ICIs, while TP53 co-mutation is the opposite. KRAS combined with SMARCA4 mutant LUAD has a poor response to non-immunotherapy and immunotherapy, and SMARCA4 mutation may be a genetic factor contributing to its poor response (**Figure 5**). The reasons for the poor efficacy of patients with KRAS-mutant NSCLC and the large interpatient variability may relate to oncogenic mechanisms and not to the function of the target itself. The key point is that there is a high degree of heterogeneity among the subtypes of KRAS mutations. Coexisting genetic events and differences in KRAS allele mutations determine different metabolic profiles and TME, both of which will produce significant differential drug sensitivities in seemingly similar tumors. Therefore, with individualized treatments for different KRAS mutant subtypes, we may eventually change the process of fatal NSCLC. In conclusion, the innovation of traditional treatment strategies and the emergence of new promising drugs may change the treatment pattern of KRAS mutant lung cancer. Yet the therapeutic strategy of KRAS gene mutation remains to be further explored.

## Author Contributions

HY provided the initial idea for this review. MS and RQ were in charge of data acquisition and drafting of the article. JR and DL revised the article. All authors read and approved the final manuscript.

## Funding

This work was supported by the National Natural Science Foundation of China (no. 81874221), Basic Public Welfare Research Project of Zhejiang Province (LGF21H160027), and Science and Technology Agency of Taizhou City (20ywa09).

## Conflict of Interest

The authors declare that the research was conducted in the absence of any commercial or financial relationships that could be construed as a potential conflict of interest.

## Publisher’s Note

All claims expressed in this article are solely those of the authors and do not necessarily represent those of their affiliated organizations, or those of the publisher, the editors and the reviewers. Any product that may be evaluated in this article, or claim that may be made by its manufacturer, is not guaranteed or endorsed by the publisher.

## Glossary

NSCLC: non-small cell lung cancer
EGFR: epidermal growth factor receptor
ALK: anaplastic lymphoma receptor tyrosine kinase
KRAS: kirsten rat sarcoma viral oncogene homolog
ROS1: proto-oncogene tyrosine-protein kinase
RET: rearranged during transfection proto-oncogene
RAS: rat sarcoma viral oncogene homolog
GTP: guanosine triphosphate
GDP: guanosine diphosphate
GEFs: guanine nucleotide exchange factors
GAPs: GTPase-activating proteins
FGFR: fibroblast growth factor receptor
HER2–4/ERBB2–4: human epidermal growth factor receptors 2–4
Raf: rat fibrosarcoma
MEK: mitogen-activated protein kinase kinase
ERK: extracellular regulated kinase
PI3K: phosphoinositide 3-kinase
Akt/PKB: protein kinase B
RASSF1: Ras association domain family 1
MAPK: mitogen-activated protein kinase
RAL: RAS-associated protein
PFS: progression-free survival
OS: overall survival
WT: wild type
DCR: disease control rate
LUAD: lung adenocarcinoma
VEGF: vascular endothelial growth factor
IL-8: CXC chemokine interleukin-8
BEV: bevacizumab
EGFR-TKIs: EGFR tyrosine kinase inhibitors
S-IIP: switch II pocket
mPFS: median progression-free survival
PD-1: programmed cell death protein 1
PD-L1: programmed cell death-ligand 1
ICIs: immune checkpoint inhibitors
ORR: objective response rate
TME: tumor microenvironment
TP53: tumor protein 53
STK11: serine-threonine kinase 11
LKB1: liver kinase B1
KL: KRAS-STK11/LKB1 co-mutant
KP: KRAS-TP53 co-mutant
AMPK: activating phosphorylation of protein kinase
TILs: tumor-infiltrating lymphocytes
IFN: interferon
IL-6: interleukin-6
KEAP1: Kelch-like ECH-associated protein 1
NFE2L2/NRF2: negative regulator of nuclear factor erythroid 2-like 2
LOF: loss of function
KLK: kallikrein-related peptidases
TCA: tricarboxylic acid
SWI/SNF: SWItch/Sucrose Non-Fermentable
SMARCA4: SWI/SNF related, matrix associated, actin dependent regulator of chromatin, subfamily A, member 4
BRG1: brahma-related gene1
KS: KRAS-SMARCA4 co-mutant

## References

[1] Siegel RL Miller KD Fuchs HE Jemal A. Cancer Statistics, 2021. CA Cancer J Clin (2021) 71:7–33. doi: 10.3322/caac.21654 33433946

[2] Chen Z Fillmore CM Hammerman PS Kim CF Wong K-K. Non-Small-Cell Lung Cancers: A Heterogeneous Set of Diseases. Nat Rev Cancer (2014) 14:535–46. doi: 10.1038/nrc3775 PMC5712844 25056707

[3] Zhou C Wu Y-L Chen G Feng J Liu X-Q Wang C. Erlotinib Versus Chemotherapy as First-Line Treatment for Patients With Advanced EGFR Mutation-Positive Non-Small-Cell Lung Cancer (OPTIMAL, CTONG-0802): A Multicentre, Open-Label, Randomised, Phase 3 Study. Lancet Oncol (2011) 12:735–42. doi: 10.1016/S1470-2045(11)70184-X 21783417

[4] Gao B Sun Y Zhang J Ren Y Fang R Han X. Spectrum of LKB1, EGFR, and KRAS Mutations in Chinese Lung Adenocarcinomas. J Thorac Oncol (2010) 5:1130–5. doi: 10.1097/JTO.0b013e3181e05016 PMC4009449 20559149

[5] Friboulet L Li N Katayama R Lee CC Gainor JF Crystal AS. The ALK Inhibitor Ceritinib Overcomes Crizotinib Resistance in Non-Small Cell Lung Cancer. Cancer Discov (2014) 4:662–73. doi: 10.1158/2159-8290.CD-13-0846 PMC4068971 24675041

[6] Solomon BJ Mok T Kim DW Wu Y-L Nakagawa K Mekhail T. First-Line Crizotinib Versus Chemotherapy in ALK-Positive Lung Cancer. N Engl J Med (2014) 371:2167–77. doi: 10.1056/NEJMoa1408440 25470694

[7] Riely GJ Marks J Pao W. KRAS Mutations in Non-Small Cell Lung Cancer. Proc Am Thorac Soc (2009) 6:201–5. doi: 10.1513/pats.200809-107LC 19349489

[8] Piva S Ganzinelli M Garassino MC Caiola E Farina G Broggini M. Across the Universe of K-RAS Mutations in Non-Small-Cell-Lung Cancer. Curr Pharm Des (2014) 20:3933–43. doi: 10.2174/13816128113196660761 24138715

[9] Xia N An J Jiang QQ Li M Tan J Hu C-p. Analysis of EGFR, EML4-ALK, KRAS, and C-MET Mutations in Chinese Lung Adenocarcinoma Patients. Exp Lung Res (2013) 39:328–35. doi: 10.3109/01902148.2013.819535 23919423

[10] Miyanaga A Shimizu K Noro R Seike M Kitamura K Kosaihira S. Activity of EGFR-Tyrosine Kinase and ALK Inhibitors for EML4-ALK-Rearranged Non-Small-Cell Lung Cancer Harbored Coexisting EGFR Mutation. BMC Cancer (2013) 13:262. doi: 10.1186/1471-2407-13-262 23714228 PMC3671182

[11] Dogan S Shen R Ang DC Johnson ML D'Angelo SP Paik PK. Molecular Epidemiology of EGFR and KRAS Mutations in 3,026 Lung Adenocarcinomas: Higher Susceptibility of Women to Smoking-Related KRAS-Mutant Cancers. Clin Cancer Res (2012) 18:6169–77. doi: 10.1158/1078-0432.CCR-11-3265 PMC3500422 23014527

[12] Ahmadzada T Kao S Reid G Boyer M Mahar A Cooper WA. An Update on Predictive Biomarkers for Treatment Selection in Non-Small Cell Lung Cancer. J Clin Med (2018) 7(6):153. doi: 10.3390/jcm7060153 PMC6025105 29914100

[13] Furugaki K Mochizuki M Kohno M Shu S Harada N Yoshimura Y. Expression of C-Terminal ALK, RET, or ROS1 in Lung Cancer Cells With or Without Fusion. BMC Cancer (2019) 19:301. doi: 10.1186/s12885-019-5527-2 30943926 PMC6446279

[14] Mazieres J Drilon A Lusque A Mhanna L Cortot AB Mezquita L. Immune Checkpoint Inhibitors for Patients With Advanced Lung Cancer and Oncogenic Driver Alterations: Results From the IMMUNOTARGET Registry. Ann Oncol (2019) 30:1321–8. doi: 10.1093/annonc/mdz167 PMC7389252 31125062

[15] Cox AD Fesik SW Kimmelman AC Luo J Der CJ. Drugging the Undruggable RAS: Mission Possible? Nat Rev Drug Discov (2014) 13:828–51. doi: 10.1038/nrd4389 PMC4355017 25323927

[16] Kranenburg O. The KRAS Oncogene: Past, Present, and Future. Biochim Biophys Acta (2005) 1756:81–2. doi: 10.1016/j.bbcan.2005.10.001 16269215

[17] Ferrer I Zugazagoitia J Herbertz S John W Paz-Ares L Schmid-Bindert G. KRAS-Mutant Non-Small Cell Lung Cancer: From Biology to Therapy. Int J Mol Sci (2020) 21(12):4325. doi: 10.3390/ijms21124325 30268480

[18] Uras IZ Moll HP Casanova E. Targeting KRAS Mutant Non-Small-Cell Lung Cancer: Past, Present and Future. Int J Mol Sci (2020) 21(12):4325. doi: 10.3390/ijms21124325 PMC7352653 32560574

[19] Barbacid M. Ras Genes. Annu Rev Biochem (1987) 56:779–827. doi: 10.1146/annurev.bi.56.070187.004023 3304147

[20] Malumbres M Barbacid M. RAS Oncogenes: The First 30 Years. Nat Rev Cancer (2003) 3:459–65. doi: 10.1038/nrc1097 12778136

[21] Karnoub AE Weinberg RA. Ras Oncogenes: Split Personalities. Nat Rev Mol Cell Biol (2008) 9:517–31. doi: 10.1038/nrm2438 PMC3915522 18568040

[22] Cox AD Der CJ. Ras History: The Saga Continues. Small GTPases (2010) 1:2–27. doi: 10.4161/sgtp.1.1.12178 21686117 PMC3109476

[23] Gibbs JB Sigal IS Poe M Scolnick EM. Intrinsic GTPase Activity Distinguishes Normal and Oncogenic Ras P21 Molecules. Proc Natl Acad Sci USA (1984) 81:5704–8 .doi: 10.1073/pnas.81.18.5704 PMC391779 6148751

[24] McGrath JP Capon DJ Goeddel DV Levinson AD. Comparative Biochemical Properties of Normal and Activated Human Ras P21 Protein. Nature (1984) 310:644–9. doi: 10.1038/310644a0 6147754

[25] Sweet RW Yokoyama S Kamata T Feramisco JR Rosenberg M Gross M. The Product of Ras Is a GTPase and the T24 Oncogenic Mutant Is Deficient in This Activity. Nature (1984) 311:273–5. doi: 10.1038/311273a0 6148703

[26] McCormick F. KRAS as a Therapeutic Target. Clin Cancer Res (2015) 21:1797–801. doi: 10.1158/1078-0432.CCR-14-2662 PMC4407814 25878360

[27] Stephen AG Esposito D Bagni RK McCormick F. Dragging Ras Back in the Ring. Cancer Cell (2014) 25:272–81. doi: 10.1016/j.ccr.2014.02.017 24651010

[28] Cromm PM Spiegel J Grossmann TN Waldmann H. Direct Modulation of Small GTPase Activity and Function. Angew Chem Int Ed Engl (2015) 54:13516–37. doi: 10.1002/anie.201504357 26470842

[29] Ostrem JM Peters U Sos ML Wells JA Shokat KM. K-Ras(G12C) Inhibitors Allosterically Control GTP Affinity and Effector Interactions. Nature (2013) 503:548–51. doi: 10.1038/nature12796 PMC4274051 24256730

[30] Xiong Y Lu J Hunter J Li L Scott D Choi HG. Covalent Guanosine Mimetic Inhibitors of G12C KRAS. ACS Med Chem Lett (2017) 8:61–6. doi: 10.1021/acsmedchemlett.6b00373 PMC5238463 28105276

[31] Zeng M Lu J Li L Feru F Quan C Gero TW. Potent and Selective Covalent Quinazoline Inhibitors of KRAS G12C. Cell Chem Biol (2017) 24:1005–1016.e3. doi: 10.1016/j.chembiol.2017.06.017 28781124

[32] Patricelli MP Janes MR Li LS Hansen R Peters U Kessler LV. Selective Inhibition of Oncogenic KRAS Output With Small Molecules Targeting the Inactive State. Cancer Discov (2016) 6:316–29. doi: 10.1158/2159-8290.CD-15-1105 26739882

[33] Roberts PJ Stinchcombe TE. KRAS Mutation: Should We Test for It, and Does It Matter? J Clin Oncol (2013) 31:1112–21. doi: 10.1200/JCO.2012.43.0454 23401440

[34] Gupta S Ramjaun AR Haiko P Wang Y Warne PH Nicke B. Binding of Ras to Phosphoinositide 3-Kinase P110alpha Is Required for Ras-Driven Tumorigenesis in Mice. Cell (2007) 129:957–68. doi: 10.1016/j.cell.2007.03.051 17540175

[35] Rodriguez-Viciana P Warne PH Khwaja A Marte BM Pappin D Das P. Role of Phosphoinositide 3-OH Kinase in Cell Transformation and Control of the Actin Cytoskeleton by Ras. Cell (1997) 89:457–67. doi: 10.1016/S0092-8674(00)80226-3 9150145

[36] van der Weyden L Adams DJ. The Ras-Association Domain Family (RASSF) Members and Their Role in Human Tumourigenesis. Biochim Biophys Acta (2007) 1776:58–85. doi: 10.1016/j.bbcan.2007.06.003 17692468 PMC2586335

[37] Lambert JM Lambert QT Reuther GW Malliri A Siderovski DP Sondek J. Tiam1 Mediates Ras Activation of Rac by a PI(3)K-Independent Mechanism. Nat Cell Biol (2002) 4:621–5. doi: 10.1038/ncb833 12134164

[38] Passiglia F Malapelle U Del Re M Righi L Pagni F Furlan D. KRAS Inhibition in Non-Small Cell Lung Cancer: Past Failures, New Findings and Upcoming Challenges. Eur J Cancer (2020) 137:57–68. doi: 10.1016/j.ejca.2020.06.023 32745965

[39] Tate JG Bamford S Jubb HC Sondka Z Beare DM Bindal N. COSMIC: The Catalogue Of Somatic Mutations In Cancer. Nucleic Acids Res (2019) 47:941–7. doi: 10.1093/nar/gky1015. PMC6323903 30371878

[40] Karachaliou N Mayo C Costa C Magrí I Gimenez-Capitan A Molina-Vila MA. KRAS Mutations in Lung Cancer. Clin Lung Cancer (2013) 14:205–14. doi: 10.1016/j.cllc.2012.09.007 23122493

[41] Liu Y Li H Zhu J Zhang Y Liu X Li R. The Prevalence and Concurrent Pathogenic Mutations of KRAS (G12C) in Northeast Chinese Non-Small-Cell Lung Cancer Patients. Cancer Manag Res (2021) 13:2447–54. doi: 10.2147/CMAR.S282617 PMC7979353 33758543

[42] Poulin EJ Bera AK Lu J Lin Y-J Strasser SD Paulo JA. Tissue-Specific Oncogenic Activity of KRAS(A146T). Cancer Discov (2019) 9:738–55. doi: 10.1158/2159-8290.CD-18-1220 PMC6548671 30952657

[43] Nadal E Chen G Prensner JR Shiratsuchi H Sam C Zhao L. KRAS-G12C Mutation Is Associated With Poor Outcome in Surgically Resected Lung Adenocarcinoma. J Thorac Oncol (2014) 9:1513–22. doi: 10.1097/JTO.0000000000000305 25170638

[44] Xu S Long BN Boris GH Chen A Ni S Kennedy MA. Structural Insight Into the Rearrangement of the Switch I Region in GTP-Bound G12A K-Ras. Acta Crystallogr D Struct Biol (2017) 73:970–84. doi: 10.1107/S2059798317015418 29199977

[45] Cruz-Migoni A Canning P Quevedo CE Bataille CJR Bery N Miller A. Structure-Based Development of New RAS-Effector Inhibitors From a Combination of Active and Inactive RAS-Binding Compounds. Proc Natl Acad Sci USA (2019) 116:2545–50. doi: 10.1073/pnas.1811360116 PMC6377466 30683716

[46] Pettersen EF Goddard TD Huang CC Couch GS Greenblatt DM Meng EC. UCSF Chimera–A Visualization System for Exploratory Research and Analysis. J Comput Chem (2004) 25:1605–12. doi: 10.1002/jcc.20084 15264254

[47] Ihle NT Byers LA Kim ES Saintigny P Lee JJ Blumenschein GR. Effect of KRAS Oncogene Substitutions on Protein Behavior: Implications for Signaling and Clinical Outcome. J Natl Cancer Inst (2012) 104:228–39. doi: 10.1093/jnci/djr523 PMC3274509 22247021

[48] Garassino MC Marabese M Rusconi P Rulli E Martelli O Farina G. Different Types of K-Ras Mutations Could Affect Drug Sensitivity and Tumour Behaviour in Non-Small-Cell Lung Cancer. Ann Oncol (2011) 22:235–7. doi: 10.1093/annonc/mdq680 21169473

[49] Jia Y Jiang T Li X Zhao C Zhang L Zhao S. Characterization of Distinct Types of KRAS Mutation and Its Impact on First-Line Platinum-Based Chemotherapy in Chinese Patients With Advanced Non-Small Cell Lung Cancer. Oncol Lett (2017) 14:6525–32. doi: 10.3892/ol.2017.7016 PMC5686437 29163686

[50] Cserepes M Ostoros G Lohinai Z Raso E Barbai T Timar J. Subtype-Specific KRAS Mutations in Advanced Lung Adenocarcinoma: A Retrospective Study of Patients Treated With Platinum-Based Chemotherapy. Eur J Cancer (2014) 50:1819–28. doi: 10.1016/j.ejca.2014.04.001 24768329

[51] Sun JM Hwang DW Ahn JS Ahn M-J Park K. Prognostic and Predictive Value of KRAS Mutations in Advanced Non-Small Cell Lung Cancer. PloS One (2013) 8:e64816. doi: 10.1371/journal.pone.0064816 23724098 PMC3665805

[52] Ghimessy AK Gellert A Schlegl E Hegedus B Raso E Barbai T. KRAS Mutations Predict Response and Outcome in Advanced Lung Adenocarcinoma Patients Receiving First-Line Bevacizumab and Platinum-Based Chemotherapy. Cancers (Basel) (2019) 11(10):1514. doi: 10.3390/cancers11101514 PMC6827133 31600989

[53] Skoulidis F Li BT Dy GK Price TJ Falchook GS Wolf J. Sotorasib for Lung Cancers With KRAS P.G12C Mutation. N Engl J Med (2021) 384:2371–81. doi: 10.1056/NEJMoa2103695 PMC9116274 34096690

[54] Jeanson A Tomasini P Souquet-Bressand M Brandone N Boucekine M Grangeon M. Efficacy of Immune Checkpoint Inhibitors in KRAS-Mutant Non-Small Cell Lung Cancer (NSCLC). J Thorac Oncol (2019) 14:1095–101. doi: 10.1016/j.jtho.2019.01.011 30738221

[55] Janne PA Smith I McWalter G Mann H Dougherty B Walker J. Impact of KRAS Codon Subtypes From a Randomised Phase II Trial of Selumetinib Plus Docetaxel in KRAS Mutant Advanced Non-Small-Cell Lung Cancer. Br J Cancer (2015) 113:199–203. doi: 10.1038/bjc.2015.215 26125448 PMC4506393

[56] Rodenhuis S Boerrigter L Top B Slebos RJ Mooi WJ Veer Lv. Mutational Activation of the K-Ras Oncogene and the Effect of Chemotherapy in Advanced Adenocarcinoma of the Lung: A Prospective Study. J Clin Oncol (1997) 15:285–91. doi: 10.1200/JCO.1997.15.1.285 8996154

[57] Schiller JH Adak S Feins RH Keller SM Fry WA Livingston RB. Lack of Prognostic Significance of P53 and K-Ras Mutations in Primary Resected Non-Small-Cell Lung Cancer on E4592: A Laboratory Ancillary Study on an Eastern Cooperative Oncology Group Prospective Randomized Trial of Postoperative Adjuvant Therapy. J Clin Oncol (2001) 19:448–57. doi: 10.1200/JCO.2001.19.2.448 11208838

[58] Eberhard DA Johnson BE Amler LC Goddard AD Heldens SL Herbst RS. Mutations in the Epidermal Growth Factor Receptor and in KRAS Are Predictive and Prognostic Indicators in Patients With Non-Small-Cell Lung Cancer Treated With Chemotherapy Alone and in Combination With Erlotinib. J Clin Oncol (2005) 23:5900–9. doi: 10.1200/JCO.2005.02.857 16043828

[59] Tsao MS Aviel-Ronen S Ding K Lau D Liu N Sakurada A. Prognostic and Predictive Importance of P53 and RAS for Adjuvant Chemotherapy in Non Small-Cell Lung Cancer. J Clin Oncol (2007) 25:5240–7. doi: 10.1200/JCO.2007.12.6953 18024870

[60] Shepherd FA Domerg C Hainaut P Jänne PA Pignon J-P Graziano S. Pooled Analysis of the Prognostic and Predictive Effects of KRAS Mutation Status and KRAS Mutation Subtype in Early-Stage Resected Non-Small-Cell Lung Cancer in Four Trials of Adjuvant Chemotherapy. J Clin Oncol (2013) 31:2173–81. doi: 10.1200/JCO.2012.48.1390 PMC4881333 23630215

[61] Metro G Chiari R Bennati C Cenci M Ricciuti B Puma F. Clinical Outcome With Platinum-Based Chemotherapy in Patients With Advanced Nonsquamous EGFR Wild-Type Non-Small-Cell Lung Cancer Segregated According to KRAS Mutation Status. Clin Lung Cancer (2014) 15:86–92. doi: 10.1016/j.cllc.2013.08.002 24139827

[62] Kranenburg O Gebbink MF Voest EE. Stimulation of Angiogenesis by Ras Proteins. Biochim Biophys Acta (2004) 1654:23–37. doi: 10.1016/j.bbcan.2003.09.004 14984765

[63] Tang Y Kim M Carrasco D Kung AL Chin L Weissleder R. *In Vivo* Assessment of RAS-Dependent Maintenance of Tumor Angiogenesis by Real-Time Magnetic Resonance Imaging. Cancer Res (2005) 65:8324–30. doi: 10.1158/0008-5472.CAN-05-0027 16166309

[64] Sparmann A Bar-Sagi D. Ras-Induced Interleukin-8 Expression Plays a Critical Role in Tumor Growth and Angiogenesis. Cancer Cell (2004) 6:447–58. doi: 10.1016/j.ccr.2004.09.028 15542429

[65] Matsuo Y Campbell PM Brekken RA Sung B Ouellette MM Fleming JB. K-Ras Promotes Angiogenesis Mediated by Immortalized Human Pancreatic Epithelial Cells Through Mitogen-Activated Protein Kinase Signaling Pathways. Mol Cancer Res (2009) 7:799–808. doi: 10.1158/1541-7786.MCR-08-0577 19509115 PMC4267726

[66] Sunaga N Imai H Shimizu K Shames DS Kakegawa S Girard L. Oncogenic KRAS-Induced Interleukin-8 Overexpression Promotes Cell Growth and Migration and Contributes to Aggressive Phenotypes of Non-Small Cell Lung Cancer. Int J Cancer (2012) 130:1733–44. doi: 10.1002/ijc.26164 PMC3374723 21544811

[67] Murillo MM Zelenay S Nye E Castellano E Lassailly F Stamp G. RAS Interaction With PI3K P110α Is Required for Tumor-Induced Angiogenesis. J Clin Invest (2014) 124:3601–11. doi: 10.1172/JCI74134 PMC4109531 25003191

[68] Al-Abd AM Alamoudi AJ Abdel-Naim AB Neamatallah TA Ashour OM. Anti-Angiogenic Agents for the Treatment of Solid Tumors: Potential Pathways, Therapy and Current Strategies - A Review. J Adv Res (2017) 8:591–605. doi: 10.1016/j.jare.2017.06.006 28808589 PMC5544473

[69] Kong DH Kim MR Jang JH Na H-J Lee S. A Review of Anti-Angiogenic Targets for Monoclonal Antibody Cancer Therapy. Int J Mol Sci (2017) 18(8):1786. doi: 10.3390/ijms18081786 PMC5578174 28817103

[70] Okada F Rak JW Croix BS Lieubeau B Kaya M Roncari L. Impact of Oncogenes in Tumor Angiogenesis: Mutant K-Ras Up-Regulation of Vascular Endothelial Growth Factor/Vascular Permeability Factor Is Necessary, But Not Sufficient for Tumorigenicity of Human Colorectal Carcinoma Cells. Proc Natl Acad Sci USA (1998) 95:3609–14. doi: 10.1073/pnas.95.7.3609 PMC19883 9520413

[71] Grunstein J Roberts WG Mathieu-Costello O Hanahan D Johnson RS. Tumor-Derived Expression of Vascular Endothelial Growth Factor Is a Critical Factor in Tumor Expansion and Vascular Function. Cancer Res (1999) 59:1592–8. 10197634

[72] Chin L Tam A Pomerantz J Wong M Holash J Bardeesy N. Essential Role for Oncogenic Ras in Tumour Maintenance. Nature (1999) 400:468–72. doi: 10.1038/22788 10440378

[73] Chaft JE Rusch V Ginsberg MS Paik PK Finley DJ Kris MG. Phase II Trial of Neoadjuvant Bevacizumab Plus Chemotherapy and Adjuvant Bevacizumab in Patients With Resectable Nonsquamous Non-Small-Cell Lung Cancers. J Thorac Oncol (2013) 8:1084–90. doi: 10.1097/JTO.0b013e31829923ec PMC4191830 23857398

[74] Metro G Chiari R Duranti S Siggillino A Fischer MJ Giannarelli D. Impact of Specific Mutant KRAS on Clinical Outcome of EGFR-TKI-Treated Advanced Non-Small Cell Lung Cancer Patients With an EGFR Wild Type Genotype. Lung Cancer (2012) 78:81–6. doi: 10.1016/j.lungcan.2012.06.005 22770374

[75] Zer A Ding K Lee SM Goss GD Seymour L Ellis PM. Pooled Analysis of the Prognostic and Predictive Value of KRAS Mutation Status and Mutation Subtype in Patients With Non-Small Cell Lung Cancer Treated With Epidermal Growth Factor Receptor Tyrosine Kinase Inhibitors. J Thorac Oncol (2016) 11:312–23. doi: 10.1016/j.jtho.2015.11.010 26749487

[76] Fiala O Pesek M Finek J Benesova L Belsanova B Minarik M. The Dominant Role of G12C Over Other KRAS Mutation Types in the Negative Prediction of Efficacy of Epidermal Growth Factor Receptor Tyrosine Kinase Inhibitors in Non-Small Cell Lung Cancer. Cancer Genet (2013) 206:26–31. doi: 10.1016/j.cancergen.2012.12.003 23313110

[77] Mao C Qiu LX Liao RY Du F-B Ding H Yang W-C. KRAS Mutations and Resistance to EGFR-TKIs Treatment in Patients With Non-Small Cell Lung Cancer: A Meta-Analysis of 22 Studies. Lung Cancer (2010) 69:272–8. doi: 10.1016/j.lungcan.2009.11.020 20022659

[78] Ni D Li X He X Zhang H Zhang J Lu S. Drugging K-Ras(G12C) Through Covalent Inhibitors: Mission Possible? Pharmacol Ther (2019) 202:1–17. doi: 10.1016/j.pharmthera.2019.06.007 31233765

[79] Janes MR Zhang J Li LS Hansen R Peters U Guo X. Targeting KRAS Mutant Cancers With a Covalent G12C-Specific Inhibitor. Cell (2018) 172:578–89.e17. doi: 10.1016/j.cell.2018.01.006 29373830

[80] Hallin J Engstrom LD Hargis L Calinisan A Aranda R Briere DM. The KRAS(G12C) Inhibitor MRTX849 Provides Insight Toward Therapeutic Susceptibility of KRAS-Mutant Cancers in Mouse Models and Patients. Cancer Discovery (2020) 10:54–71. doi: 10.1158/2159-8290.CD-19-1167 31658955 PMC6954325

[81] Nagasaka M Li Y Sukari A Ou S-HI Al-Hallak MN Azmi AS. KRAS G12C Game of Thrones, Which Direct KRAS Inhibitor Will Claim the Iron Throne? Cancer Treat Rev (2020) 84:101974. doi: 10.1016/j.ctrv.2020.101974 32014824 PMC7041424

[82] D'Incecco A Andreozzi M Ludovini V Rossi E Capodanno A Landi L. PD-1 and PD-L1 Expression in Molecularly Selected Non-Small-Cell Lung Cancer Patients. Br J Cancer (2015) 112:95–102. doi: 10.1038/bjc.2014.555 25349974 PMC4453606

[83] Dong ZY Zhong WZ Zhang XC Su J Xie Z Liu S-Y. Potential Predictive Value of TP53 and KRAS Mutation Status for Response to PD-1 Blockade Immunotherapy in Lung Adenocarcinoma. Clin Cancer Res (2017) 23:3012–24. doi: 10.1158/1078-0432.CCR-16-2554 28039262

[84] Lee M-H Yanagawa J Li R Walser TC Krysan K Wang G. Increased PD-L1 Expression in KRAS Mutated Premalignant Human Bronchial Epithelial Cells Is Enhanced by LKB1 Loss and Mediated by ERK Activation. J ImmunoTher Cancer (2015) 3(Suppl 2):P305. doi: 10.1186/2051-1426-3-S2-P305

[85] Chen N Fang W Lin Z Peng P Wang J Zhan J. KRAS Mutation-Induced Upregulation of PD-L1 Mediates Mmune Escape in Human Lung Adenocarcinoma. Cancer Immunol Immunother (2017) 66:1175–87. doi: 10.1007/s00262-017-2005-z PMC5579171 28451792

[86] Wu ZX Liu Z Jiang HL Pan H-M Han W-D. Circulating Tumor Cells Predict Survival Benefit From Chemotherapy in Patients With Lung Cancer. Oncotarget (2016) 7:67586–96. doi: 10.18632/oncotarget.11707 PMC5341898 27588489

[87] Muinelo-Romay L Vieito M Abalo A Nocelo MA Barón F Anido U. Evaluation of Circulating Tumor Cells and Related Events as Prognostic Factors and Surrogate Biomarkers in Advanced NSCLC Patients Receiving First-Line Systemic Treatment. Cancers (Basel) (2014) 6:153–65. doi: 10.3390/cancers6010153 PMC3980598 24452143

[88] Guibert N Delaunay M Lusque A Boubekeur N Rouquette I Clermont E. PD-L1 Expression in Circulating Tumor Cells of Advanced Non-Small Cell Lung Cancer Patients Treated With Nivolumab. Lung Cancer (2018) 120:108–12. doi: 10.1016/j.lungcan.2018.04.001 29748004

[89] Wang Y Kim TH Fouladdel S Zhang Z Soni P Qin A. PD-L1 Expression in Circulating Tumor Cells Increases During Radio(chemo)therapy and Indicates Poor Prognosis in Non-Small Cell Lung Cancer. Sci Rep (2019) 9:566. doi: 10.1038/s41598-018-36096-7 30679441 PMC6345864

[90] Nicolazzo C Raimondi C Mancini M Caponnetto S Gradilone A Gandini O. Monitoring PD-L1 Positive Circulating Tumor Cells in Non-Small Cell Lung Cancer Patients Treated With the PD-1 Inhibitor Nivolumab. Sci Rep (2016) 6:31726. doi: 10.1038/srep31726 27553175 PMC4995431

[91] Tashima Y Kuwata T Yoneda K Hirai A Mori M Kanayama M. Prognostic Impact of PD-L1 Expression in Correlation With Neutrophil-to-Lymphocyte Ratio in Squamous Cell Carcinoma of the Lung. Sci Rep (2020) 10:1243. doi: 10.1038/s41598-019-57321-x 31988315 PMC6985257

[92] Zugazagoitia J Gupta S Liu Y Fuhrman K Gettinger S Herbst RS. Biomarkers Associated With Beneficial PD-1 Checkpoint Blockade in Non-Small Cell Lung Cancer (NSCLC) Identified Using High-Plex Digital Spatial Profiling. Clin Cancer Res (2020) 26:4360–8. doi: 10.1158/1078-0432.CCR-20-0175 PMC7442721 32253229

[93] Mayoux M Roller A Pulko V Sammicheli S Chen S Sum E. Dendritic Cells Dictate Responses to PD-L1 Blockade Cancer Immunotherapy. Sci Transl Med (2020) 12(534):eaav7431. doi: 10.1126/scitranslmed.aav7431 32161104

[94] Liu Y Zugazagoitia J Ahmed FS Henick BS Gettinger SN Herbst RS. Immune Cell PD-L1 Colocalizes With Macrophages and Is Associated With Outcome in PD-1 Pathway Blockade Therapy. Clin Cancer Res (2020) 26:970–7. doi: 10.1158/1078-0432.CCR-19-1040 PMC7024671 31615933

[95] O'Callaghan DS Rexhepaj E Gately K Coate L Delaney D O'Donnell DM. Tumour Islet Foxp3+ T-Cell Infiltration Predicts Poor Outcome in Nonsmall Cell Lung Cancer. Eur Respir J (2015) 46:1762–72. doi: 10.1183/13993003.00176-2014 26541534

[96] Swanton C. Take Lessons From Cancer Evolution to the Clinic. Nature (2020) 581:382–3. doi: 10.1038/d41586-020-01347-z 32461644

[97] Poutahidis T Erdman SE. Commensal Bacteria Modulate the Tumor Microenvironment. Cancer Lett (2016) 380:356–8. doi: 10.1016/j.canlet.2015.12.028 PMC4942371 26739062

[98] Mager LF Burkhard R Pett N Cooke NCA Brown K Ramay H. Microbiome-Derived Inosine Modulates Response to Checkpoint Inhibitor Immunotherapy. Science (2020) 369:1481–9. doi: 10.1126/science.abc3421 32792462

[99] Yang W Yu T Huang X Bilotta AJ Xu L Lu Y. Intestinal Microbiota-Derived Short-Chain Fatty Acids Regulation of Immune Cell IL-22 Production and Gut Immunity. Nat Commun (2020) 11:4457. doi: 10.1038/s41467-020-18262-6 32901017 PMC7478978

[100] Ohtani N. Microbiome and Cancer. Semin Immunopathol (2015) 37:65–72. doi: 10.1007/s00281-014-0457-1 25404117

[101] Dibra D Xia X Mitra A Cutrera JJ Lozano G Li S. Mutant P53 in Concert With an Interleukin-27 Receptor Alpha Deficiency Causes Spontaneous Liver Inflammation, Fibrosis, and Steatosis in Mice. Hepatology (2016) 63:1000–12. doi: 10.1002/hep.28379 PMC4764463 26637970

[102] Pylayeva-Gupta Y Grabocka E Bar-Sagi D. RAS Oncogenes: Weaving a Tumorigenic Web. Nat Rev Cancer (2011) 11:761–74. doi: 10.1038/nrc3106 PMC3632399 21993244

[103] Castellano E Downward J. RAS Interaction With PI3K: More Than Just Another Effector Pathway. Genes Cancer (2011) 2:261–74. doi: 10.1177/1947601911408079 PMC3128635 21779497

[104] Smith MJ Ikura M. Integrated RAS Signaling Defined by Parallel NMR Detection of Effectors and Regulators. Nat Chem Biol (2014) 10:223–30. doi: 10.1038/nchembio.1435 24441586

[105] Janakiraman M Vakiani E Zeng Z Pratilas CA Taylor BS Chitale D. Genomic and Biological Characterization of Exon 4 KRAS Mutations in Human Cancer. Cancer Res (2010) 70:5901–11. doi: 10.1158/0008-5472.CAN-10-0192 PMC2943514 20570890

[106] Caiola E Brunelli L Marabese M Broggini M Lupi M Pastorelli R. Different Metabolic Responses to PI3K Inhibition in NSCLC Cells Harboring Wild-Type and G12C Mutant KRAS. Oncotarget (2016) 7:51462–72. doi: 10.18632/oncotarget.9849 PMC5239488 27283493

[107] Liu C Zheng S Jin R Wang X Wang F Zang R. The Superior Efficacy of Anti-PD-1/PD-L1 Immunotherapy in KRAS-Mutant Non-Small Cell Lung Cancer That Correlates With an Inflammatory Phenotype and Increased Immunogenicity. Cancer Lett (2020) 470:95–105. doi: 10.1016/j.canlet.2019.10.027 31644929

[108] Nieto P Ambrogio C Esteban-Burgos L Gómez-López G Blasco MT Yao Z. A Braf Kinase-Inactive Mutant Induces Lung Adenocarcinoma. Nature (2017) 548:239–43. doi: 10.1038/nature23297 PMC5648056 28783725

[109] Stephens P Hunter C Bignell G Edkins S Davies H Teague J. Lung Cancer: Intragenic ERBB2 Kinase Mutations in Tumours. Nature (2004) 431:525–6. doi: 10.1038/431525b 15457249

[110] Skoulidis F Goldberg ME Greenawalt DM Hellmann MD Awad MM Gainor JF. STK11/LKB1 Mutations and PD-1 Inhibitor Resistance in KRAS-Mutant Lung Adenocarcinoma. Cancer Discov (2018) 8:822–35. doi: 10.1158/2159-8290.CD-18-0099 PMC6030433 29773717

[111] Alessi JV Ricciuti B Spurr LF Gupta H Li YY Glass C. SMARCA4 and Other SWItch/Sucrose NonFermentable Family Genomic Alterations in NSCLC: Clinicopathologic Characteristics and Outcomes to Immune Checkpoint Inhibition. J Thorac Oncol (2021) 16:1176–87. doi: 10.1016/j.jtho.2021.03.024 33845210

[112] Liu L Ahmed T Petty WJ Grant S Ruiz J Lycan TW. SMARCA4 Mutations in KRAS-Mutant Lung Adenocarcinoma: A Multi-Cohort Analysis. Mol Oncol (2021) 15:462–72. doi: 10.1002/1878-0261.12831 PMC7858279 33107184

[113] Skoulidis F Byers LA Diao L Papadimitrakopoulou VA Tong P Izzo J. Co-Occurring Genomic Alterations Define Major Subsets of KRAS-Mutant Lung Adenocarcinoma With Distinct Biology, Immune Profiles, and Therapeutic Vulnerabilities. Cancer Discov (2015) 5:860–77. doi: 10.1158/2159-8290.CD-14-1236 PMC4527963 26069186

[114] Shackelford DB Shaw RJ. The LKB1-AMPK Pathway: Metabolism and Growth Control in Tumour Suppression. Nat Rev Cancer (2009) 9:563–75. doi: 10.1038/nrc2676 PMC2756045 19629071

[115] Koyama S Akbay EA Li YY Aref AR Skoulidis F Herter-Sprie GS. STK11/LKB1 Deficiency Promotes Neutrophil Recruitment and Proinflammatory Cytokine Production to Suppress T-Cell Activity in the Lung Tumor Microenvironment. Cancer Res (2016) 76:999–1008. doi: 10.1158/0008-5472.CAN-15-1439 26833127 PMC4775354

[116] Kadara H Choi M Zhang J Parra ER Rodriguez-Canales J Gaffney SG. Whole-Exome Sequencing and Immune Profiling of Early-Stage Lung Adenocarcinoma With Fully Annotated Clinical Follow-Up. Ann Oncol (2017) 28:75–82. doi: 10.1093/annonc/mdw436 27687306 PMC5982809

[117] La Fleur L Falk-Sorqvist E Smeds P Berglund A Sundström M Mattsson JS. Mutation Patterns in a Population-Based Non-Small Cell Lung Cancer Cohort and Prognostic Impact of Concomitant Mutations in KRAS and TP53 or STK11. Lung Cancer (2019) 130:50–8. doi: 10.1016/j.lungcan.2019.01.003 30885352

[118] Schabath MB Welsh EA Fulp WJ Chen L Teer JK Thompson ZJ. Differential Association of STK11 and TP53 With KRAS Mutation-Associated Gene Expression, Proliferation and Immune Surveillance in Lung Adenocarcinoma. Oncogene (2016) 35:3209–16. doi: 10.1038/onc.2015.375 PMC4837098 26477306

[119] Skoulidis F Heymach JV. Co-Occurring Genomic Alterations in Non-Small-Cell Lung Cancer Biology and Therapy. Nat Rev Cancer (2019) 19:495–509. doi: 10.1038/s41568-019-0179-8 31406302 PMC7043073

[120] Chen X Su C Ren S Zhou C Jiang T. Pan-Cancer Analysis of KEAP1 Mutations as Biomarkers for Immunotherapy Outcomes. Ann Transl Med (2020) 8:141. doi: 10.21037/atm.2019.11.52 32175433 PMC7048975

[121] Berger AH Brooks AN Wu X Shrestha Y Chouinard C Piccioni F. High-Throughput Phenotyping of Lung Cancer Somatic Mutations. Cancer Cell (2016) 30:214–28. doi: 10.1016/j.ccell.2016.06.022 PMC5003022 27478040

[122] Cancer Genome Atlas Research Network. Comprehensive Molecular Profiling of Lung Adenocarcinoma. Nature (2014) 511:543–50. doi: 10.1038/nature13385 PMC4231481 25079552

[123] Singh A Misra V Thimmulappa RK Lee H Ames S Hoque MO. Dysfunctional KEAP1-NRF2 Interaction in Non-Small-Cell Lung Cancer. PloS Med (2006) 3:e420. doi: 10.1371/journal.pmed.0030420 17020408 PMC1584412

[124] Romero R Sayin VI Davidson SM Bauer MR Singh SX LeBoeuf SE. Keap1 Loss Promotes Kras-Driven Lung Cancer and Results in Dependence on Glutaminolysis. Nat Med (2017) 23:1362–8. doi: 10.1038/nm.4407 PMC5677540 28967920

[125] Bauer AK Cho HY Miller-Degraff L Walker C Helms K Fostel J. Targeted Deletion of Nrf2 Reduces Urethane-Induced Lung Tumor Development in Mice. PloS One (2011) 6:e26590. doi: 10.1371/journal.pone.0026590 22039513 PMC3198791

[126] DeNicola GM Karreth FA Humpton TJ Gopinathan A Wei C Frese K. Oncogene-Induced Nrf2 Transcription Promotes ROS Detoxification and Tumorigenesis. Nature (2011) 475:106–9. doi: 10.1038/nature10189 PMC3404470 21734707

[127] Satoh H Moriguchi T Takai J Nilsson JA Lindahl P Bergo MO. Nrf2 Prevents Initiation But Accelerates Progression Through the Kras Signaling Pathway During Lung Carcinogenesis. Cancer Res (2013) 73:4158–68. doi: 10.1158/0008-5472.CAN-12-4499 23610445

[128] Sayin VI Ibrahim MX Larsson E Nilsson JA Lindahl P Bergo MO. Antioxidants Accelerate Lung Cancer Progression in Mice. Sci Transl Med (2014) 6:221ra15. doi: 10.1126/scitranslmed.3007653 24477002

[129] Chio IIC Jafarnejad SM Ponz-Sarvise M Park Y Rivera K Palm W. NRF2 Promotes Tumor Maintenance by Modulating mRNA Translation in Pancreatic Cancer. Cell (2016) 166:963–76. doi: 10.1016/j.cell.2016.06.056 PMC5234705 27477511

[130] Satoh H Moriguchi T Saigusa D Baird L Yu L Rokutan H. NRF2 Intensifies Host Defense Systems to Prevent Lung Carcinogenesis, But After Tumor Initiation Accelerates Malignant Cell Growth. Cancer Res (2016) 76:3088–96. doi: 10.1158/0008-5472.CAN-15-1584 27020858

[131] Kerr EM Gaude E Turrell FK Frezza C Martins CP. Mutant Kras Copy Number Defines Metabolic Reprogramming and Therapeutic Susceptibilities. Nature (2016) 531:110–3. doi: 10.1038/nature16967 PMC4780242 26909577

[132] Cancer Genome Atlas Research Network.Comprehensive Genomic Characterization of Squamous Cell Lung Cancers. Nature (2012) 489:519–25. doi: 10.1038/nature11404 PMC3466113 22960745

[133] Jaramillo MC Zhang DD. The Emerging Role of the Nrf2-Keap1 Signaling Pathway in Cancer. Genes Dev (2013) 27:2179–91. doi: 10.1101/gad.225680.113 PMC3814639 24142871

[134] Konstantinopoulos PA Spentzos D Fountzilas E Francoeur N Sanisetty S Grammatikos AP. Keap1 Mutations and Nrf2 Pathway Activation in Epithelial Ovarian Cancer. Cancer Res (2011) 71:5081–9. doi: 10.1158/0008-5472.CAN-10-4668 21676886

[135] Shibata T Kokubu A Gotoh M Ojima H Ohta T Yamamoto M. Genetic Alteration of Keap1 Confers Constitutive Nrf2 Activation and Resistance to Chemotherapy in Gallbladder Cancer. Gastroenterology (2008) 135:1358–1368, 1368.e1-4. doi: 10.1053/j.gastro.2008.06.082 18692501

[136] Kim YR Oh JE Kim MS Kang MR Park SW Han JY. Oncogenic NRF2 Mutations in Squamous Cell Carcinomas of Oesophagus and Skin. J Pathol (2010) 220:446–51. doi: 10.1002/path.2653 19967722

[137] Sato Y Yoshizato T Shiraishi Y Maekawa S Okuno Y Kamura T. Integrated Molecular Analysis of Clear-Cell Renal Cell Carcinoma. Nat Genet (2013) 45:860–7. doi: 10.1038/ng.2699 23797736

[138] Fabrizio FP Costantini M Copetti M la Torre A Sparaneo A Fontana A. Keap1/Nrf2 Pathway in Kidney Cancer: Frequent Methylation of KEAP1 Gene Promoter in Clear Renal Cell Carcinoma. Oncotarget (2017) 8:11187–98. doi: 10.18632/oncotarget.14492 PMC5355256 28061437

[139] Muscarella LA Barbano R D'Angelo V Copetti M Coco M Balsamo T. Regulation of KEAP1 Expression by Promoter Methylation in Malignant Gliomas and Association With Patient's Outcome. Epigenetics (2011) 6:317–25. doi: 10.4161/epi.6.3.14408 PMC3092680 21173573

[140] Hanada N Takahata T Zhou Q Ye X Sun R Itoh J. Methylation of the KEAP1 Gene Promoter Region in Human Colorectal Cancer. BMC Cancer (2012) 12:66. doi: 10.1186/1471-2407-12-66 22325485 PMC3296656

[141] Goldstein LD Lee J Gnad F Klijn C Schaub A Reeder J. Recurrent Loss of NFE2L2 Exon 2 Is a Mechanism for Nrf2 Pathway Activation in Human Cancers. Cell Rep (2016) 16:2605–17. doi: 10.1016/j.celrep.2016.08.010 27568559

[142] Krall EB Wang B Munoz DM Ilic N Raghavan S Niederst MJ. KEAP1 Loss Modulates Sensitivity to Kinase Targeted Therapy in Lung Cancer. Elife (2017) 6:e33173. doi: 10.7554/eLife.33173 28145866 PMC5305212

[143] Galan-Cobo A Sitthideatphaiboon P Qu X Poteete A Pisegna MA Tong P. LKB1 and KEAP1/NRF2 Pathways Cooperatively Promote Metabolic Reprogramming With Enhanced Glutamine Dependence in KRAS-Mutant Lung Adenocarcinoma. Cancer Res (2019) 79:3251–67. doi: 10.1158/0008-5472.CAN-18-3527 PMC6606351 31040157

[144] Singh A Daemen A Nickles D Jeon S-M Foreman O Sudini K. NRF2 Activation Promotes Aggressive Lung Cancer and Associates With Poor Clinical Outcomes. Clin Cancer Res (2021) 27:877–88. doi: 10.1158/1078-0432.CCR-20-1985 PMC10867786 33077574

[145] Arbour KC Jordan E Kim HR Dienstag J Yu HA Sanchez-Vega F. Effects of Co-Occurring Genomic Alterations on Outcomes in Patients With KRAS-Mutant Non-Small Cell Lung Cancer. Clin Cancer Res (2018) 24:334–40. doi: 10.1158/1078-0432.CCR-17-1841 PMC5771996 29089357

[146] Negrao MV Lam VK Reuben A Rubin ML Landry LL Roarty EB. PD-L1 Expression, Tumor Mutational Burden, and Cancer Gene Mutations Are Stronger Predictors of Benefit From Immune Checkpoint Blockade Than HLA Class I Genotype in Non-Small Cell Lung Cancer. J Thorac Oncol (2019) 14:1021–31. doi: 10.1016/j.jtho.2019.02.008 30780001

[147] Jeong Y Hellyer JA Stehr H Hoang NT Niu X Das M. Role of KEAP1/NFE2L2 Mutations in the Chemotherapeutic Response of Patients With Non-Small Cell Lung Cancer. Clin Cancer Res (2020) 26:274–81. doi: 10.1158/1078-0432.CCR-19-1237 PMC6942632 31548347

[148] Wilson BG Roberts CW. SWI/SNF Nucleosome Remodellers and Cancer. Nat Rev Cancer (2011) 11:481–92. doi: 10.1038/nrc3068 21654818

[149] Becker PB Workman JL. Nucleosome Remodeling and Epigenetics. Cold Spring Harb Perspect Biol (2013) 5(9):a017905. doi: 10.1101/cshperspect.a017905 24003213 PMC3753709

[150] Kadoch C Hargreaves DC Hodges C Elias L Ho L Ranish J. Proteomic and Bioinformatic Analysis of Mammalian SWI/SNF Complexes Identifies Extensive Roles in Human Malignancy. Nat Genet (2013) 45:592–601. doi: 10.1038/ng.2628 23644491 PMC3667980

[151] Helming KC Wang X Roberts CWM. Vulnerabilities of Mutant SWI/SNF Complexes in Cancer. Cancer Cell (2014) 26:309–17. doi: 10.1016/j.ccr.2014.07.018 PMC4159614 25203320

[152] Imielinski M Berger AH Hammerman PS Hernandez B Pugh TJ Hodis E. Mapping the Hallmarks of Lung Adenocarcinoma With Massively Parallel Sequencing. Cell (2012) 150:1107–20. doi: 10.1016/j.cell.2012.08.029 PMC3557932 22980975

[153] Hodges HC Stanton BZ Cermakova K Chang C-Y Miller EL Kirkland JG. Dominant-Negative SMARCA4 Mutants Alter the Accessibility Landscape of Tissue-Unrestricted Enhancers. Nat Struct Mol Biol (2018) 25:61–72. doi: 10.1038/s41594-017-0007-3 29323272 PMC5909405

[154] Shain AH Pollack JR. The Spectrum of SWI/SNF Mutations, Ubiquitous in Human Cancers. PloS One (2013) 8:e55119. doi: 10.1371/journal.pone.0055119 23355908 PMC3552954

[155] Rodriguez-Nieto S Cañada A Pros E Pinto AI Torres-Lanzas J Lopez-Rios F. Massive Parallel DNA Pyrosequencing Analysis of the Tumor Suppressor BRG1/SMARCA4 in Lung Primary Tumors. Hum Mutat (2011) 32:E1999–2017. doi: 10.1002/humu.21415 21280140

[156] Orvis T Hepperla A Walter V Song S Simon J Parker J. BRG1/SMARCA4 Inactivation Promotes Non-Small Cell Lung Cancer Aggressiveness by Altering Chromatin Organization. Cancer Res (2014) 74:6486–98. doi: 10.1158/0008-5472.CAN-14-0061 PMC4233181 25115300

[157] Bell EH Chakraborty AR Mo X Liu Z Shilo K Kirste S. SMARCA4/BRG1 Is a Novel Prognostic Biomarker Predictive of Cisplatin-Based Chemotherapy Outcomes in Resected Non-Small Cell Lung Cancer. Clin Cancer Res (2016) 22:2396–404. doi: 10.1158/1078-0432.CCR-15-1468 PMC4867280 26671993

[158] Dagogo-Jack I Schrock AB Kem M Jessop N Lee J Ali SM. Clinicopathologic Characteristics of BRG1-Deficient NSCLC. J Thorac Oncol (2020) 15:766–76. doi: 10.1016/j.jtho.2020.01.002 31988001

[159] Gupta M Concepcion CP Fahey CG Keshishian H Bhutkar A Brainson CF. BRG1 Loss Predisposes Lung Cancers to Replicative Stress and ATR Dependency. Cancer Res (2020) 80:3841–54. doi: 10.1158/0008-5472.CAN-20-1744 PMC7501156 32690724

[160] Lissanu Deribe Y Sun Y Terranova C Khan F Martinez-Ledesma J Gay J. Mutations in the SWI/SNF Complex Induce a Targetable Dependence on Oxidative Phosphorylation in Lung Cancer. Nat Med (2018) 24:1047–57. doi: 10.1038/s41591-018-0019-5 PMC6650267 29892061

[161] Schoenfeld AJ Bandlamudi C Lavery JA Montecalvo J Namakydoust A Rizvi H. The Genomic Landscape of SMARCA4 Alterations and Associations With Outcomes in Patients With Lung Cancer. Clin Cancer Res (2020) 26:5701–8. doi: 10.1158/1078-0432.CCR-20-1825 PMC7641983 32709715

[162] Xue Y Meehan B Fu Z Wang XQD Fiset PO Rieker R. SMARCA4 Loss Is Synthetic Lethal With CDK4/6 Inhibition in Non-Small Cell Lung Cancer. Nat Commun (2019) 10:557. doi: 10.1038/s41467-019-08380-1 30718506 PMC6362083

